# Biofabrication of nanoparticles: sources, synthesis, and biomedical applications

**DOI:** 10.3389/fbioe.2023.1159193

**Published:** 2023-05-02

**Authors:** Deepak Kulkarni, Rushikesh Sherkar, Chaitali Shirsathe, Rushikesh Sonwane, Nikita Varpe, Santosh Shelke, Mahesh P. More, Sagar R. Pardeshi, Gargee Dhaneshwar, Vijayabhaskarreddy Junnuthula, Sathish Dyawanapelly

**Affiliations:** ^1^ Department of Pharmaceutics, Srinath College of Pharmacy, Aurangabad, Maharashtra, India; ^2^ Department of Pharmaceutics, Dr Rajendra Gode College of Pharmacy, Malkapur, Buldana, India; ^3^ Department of Pharmaceutics, St John Institute of Pharmacy and Research, Palghar, India; ^4^ MET’s Institute of Pharmacy, Nashik, Maharashtra, India; ^5^ Drug Research Program, Faculty of Pharmacy, University of Helsinki, Helsinki, Finland; ^6^ Department of Pharmaceutical Sciences and Technology, Institute of Chemical Technology, Mumbai, India

**Keywords:** biogenic synthesis, green synthesis, nanoparticles, microorganisms, plant extracts, biomedical applications, natural sources, metallic nanoparticles

## Abstract

Nanotechnology is an emerging applied science delivering crucial human interventions. Biogenic nanoparticles produced from natural sources have received attraction in recent times due to their positive attributes in both health and the environment. It is possible to produce nanoparticles using various microorganisms, plants, and marine sources. The bioreduction mechanism is generally employed for intra/extracellular synthesis of biogenic nanoparticles. Various biogenic sources have tremendous bioreduction potential, and capping agents impart stability. The obtained nanoparticles are typically characterized by conventional physical and chemical analysis techniques. Various process parameters, such as sources, ions, and temperature incubation periods, affect the production process. Unit operations such as filtration, purification, and drying play a role in the scale-up setup. Biogenic nanoparticles have extensive biomedical and healthcare applications. In this review, we summarized various sources, synthetic processes, and biomedical applications of metal nanoparticles produced by biogenic synthesis. We highlighted some of the patented inventions and their applications. The applications range from drug delivery to biosensing in various therapeutics and diagnostics. Although biogenic nanoparticles appear to be superior to their counterparts, the molecular mechanism degradation pathways, kinetics, and biodistribution are often missing in the published literature, and scientists should focus more on these aspects to move them from the bench side to clinics.

## Graphical Abstract

**Figure:**
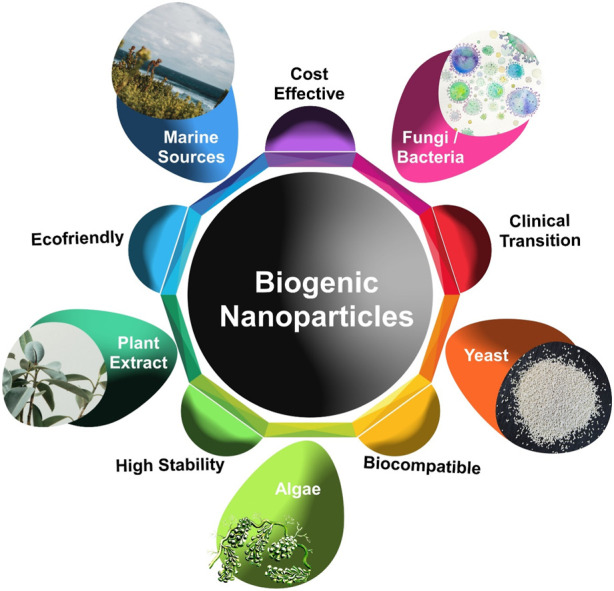
Sources for biogenic synthesis: The synthesis sources include but are not limited to bacteria, fungi, algae, yeasts, marine and plant sources.

## 1 Introduction

Green nanoparticles, which are produced utilizing sustainable methods and materials, provide several advantages over conventional nanoparticles. These benefits include biocompatibility, improved properties, cost-effectiveness, environmental sustainability, and sustainable development. Green nanoparticles are environmentally friendly since they are often produced using nontoxic, biodegradable, and renewable materials (Parveen et al., 2016). Synthetic methods for nanoparticle synthesis are frequently linked to many challenges, including the production of hazardous byproducts, instability issues, high prices, and major environmental concerns (Ashique et al., 2022). Green synthesis-based metal nanoparticles have received greater attention due to their unique physicochemical properties (Anandalakshmi, 2021; Tade et al., 2020).

The conventional methods of synthesizing nanoparticles have several limitations, such as the use of hazardous chemicals, high energy requirements, and high costs. In contrast, biogenic nanoparticle synthesis is a green and cost-effective alternative that utilizes natural biological systems such as plants, animals, fungi, and bacteria to produce nanoparticles. The green synthesis approach has an advantage over traditional synthetic processes in terms of eco-friendliness and biocompatibility. Plant extracts contain phytochemicals that are essential for the synthesis of nanoparticles and for enhancing their bioactivity. These phytochemicals include terpenoids, ketones, flavonoids, aldehydes, amides, and carboxylic acids. This approach is broadly termed “green technology,” which is advantageous and eco-friendly in comparison to physical or chemical methods (Singh et al., 2021).

Biogenic nanotechnology is the combination of biology and material science. Nanoparticles represent a fundamentally practical platform, exhibiting special qualities with potentially broad applicability. The unique qualities and utility of nanoparticles result from several factors, including their size similarity to biomolecules such as proteins and polynucleic acids (De et al., 2008). The biogenic method for synthesizing nanoparticles results in particles with good polydispersity, diameters, and stability (Ingale and Chaudhari, 2013).

An expanding number of microorganisms are employed to produce nanosized particles (Chugh et al., 2021). There are many distinct types of microorganisms, and they all react with metal precursors in somewhat different ways to make nanoparticles. Despite the unique reaction mechanism of each biogenic material, they all essentially function in the same way, leading to the production of the desired nanostructures in a “complex broth” (Srivastava et al., 2015). In addition to the characterization of these nanosystems, the focus must be placed on the extraction and identification of the relevant biomolecules to use them as prototypes for the synthesis of nanomaterials, or more accurately, “biomimetic materials” (Kim et al., 2017). Some biogenic techniques, although still in their infancy, have developed into biomimetic approaches, giving rise to sophisticated bionano hybrid materials, self-assembled functional materials, and enhanced biomedical applications (Srivastava et al., 2015).

**FIGURE 1:**
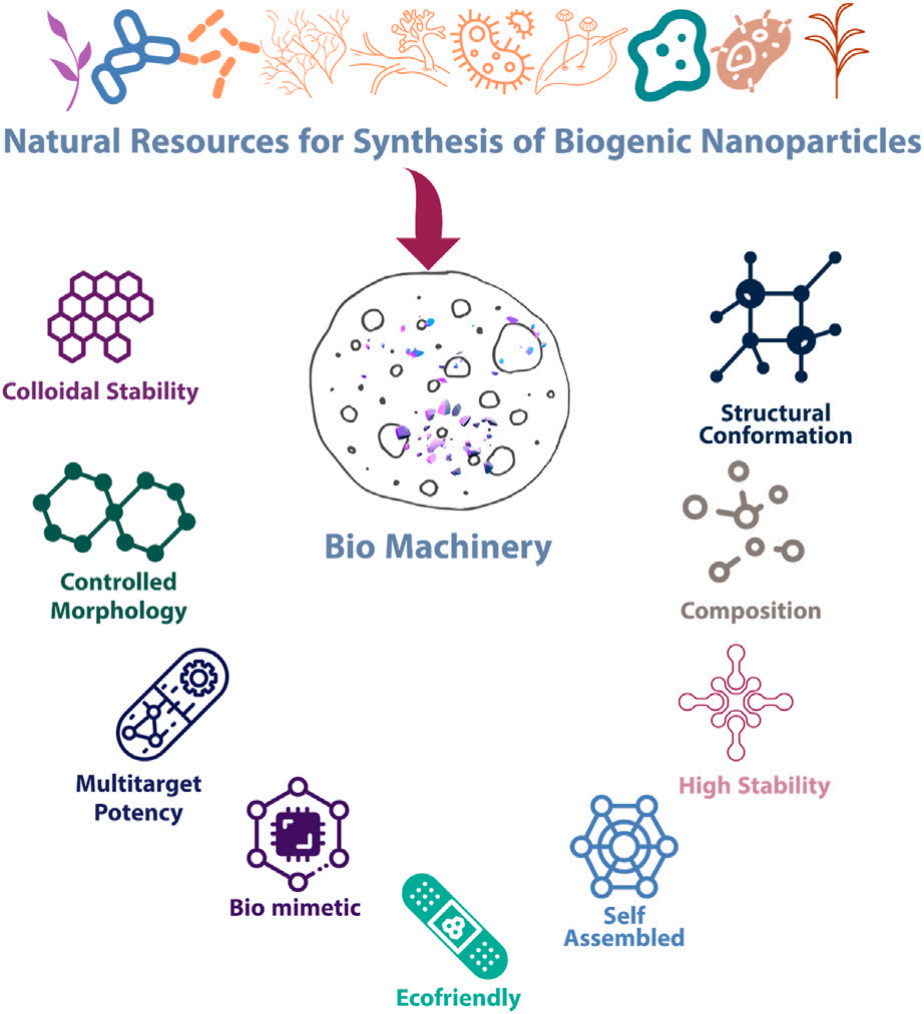
Salient features and properties of biogenic nanoparticles.

The employment of eco-friendly procedures, such as the usage of biopolymers, plant extracts, and biomolecules, has drawn growing interest to achieve this goal (Ahmed et al., 2016). They are ideal reagents because they satisfy the criteria for biocompatibility and accessibility and function in a variety of ways, such as capping, reducing, and shape-modulating agents. The various advantages of biogenic synthesis and the importance of biogenic synthesis are illustrated in Figure 1. Green chemistry is the use of chemicals to help prevent pollution in particular fields such as clean analytical methods, environmentally friendly analytical chemistry, and green analytical chemistry. The fact that green synthesis is biocompatible, inert, and harmless to the environment makes it a suitable method for creating nanoparticles (Scala et al., 2022).

The synthesis process provides eco-friendly nanoparticles with greater stability, clinical adaptability, and biocompatibility. Organisms have developed the ability to survive in environments with high metal concentrations. These organisms may alter their chemical nature and reduce their toxicity. The fabrication of nanoparticles is an outcome of a resistance mechanism of an organism to a certain metal (Dhuldhaj and Pandya, 2021). The synthesis of biogenic metallic nanoparticles is categorized into two types. 1) Biodegradation: Chemical reduction through biological means can generate more stable metal ions, which is achieved through dissimilatory metal reduction. The enzyme undergoes oxidation, whereas the metal ion undergoes reduction. The metallic nanoparticles obtained are extracted safely and subjected to further evaluations. 2) Biosorption: The metal ions present in an aqueous or soil sample become attached to the organism itself. Peptides or cell walls bonded with metal ions are obtained by some bacteria, fungi, and plants (Mittal et al., 2021). These peptides stabilize the nanoparticles. Several variables influence the selection of biological approaches for nanoparticle synthesis and creation. The most crucial variable is the form of the metal nanoparticle to be generated. The choice of organism remains limited due to the occurrence of resistance against metals (Patil et al. 2019a; Patil et al., 2017).

Recent advances in nanotechnology are expected to demonstrate a considerable impact on the progress of novel therapeutic strategies. The ability to obtain nanoparticles in the protein size domain results in a variety of biomedical applications, as they can stimulate response and improve desired therapeutic effects with minimum undesirable effects (Gupta and Gupta, 2005). Therapy for various diseases and disorders includes a variety of synthetic medicines that have demonstrated promising effects. Synthetic drug usage is linked to a variety of adverse effects, and scientists have taken a soft turn toward the use of phytochemicals, which have few side effects (BhattacharyaSoares et al., 2022). Despite various advantages, green nanotechnology has some challenges. Scale-up is one of the major challenges; currently, the production of green nanomaterials is limited to laboratory-scale experiments, and it is difficult to translate these findings into large-scale manufacturing processes (Gacem et al., 2022). Identifying the most suitable bioresource for producing the desired nanoparticles is the main challenge. Purification of nanoparticles from raw biological components after production is also a serious obstacle (Nethi et al., 2021).

This article will provide a better understanding of green technology in the fabrication of nanoparticles using bacteria, algae, fungi, yeasts, and plants for potential biomedical uses.

## 2 Sources for the synthesis of biogenic nanoparticles

The synthesis of nanoplatforms by physical and chemical methods demands highly toxic reducing agents, high radiation, and stabilizing agents (Hebbalalu et al., 2013). These can be harmful to nature as well as human life. Therefore, researchers are now interested in green synthesis for sustained and eco-friendly approaches for the synthesis of metallic and metal oxide nanoparticles (Priya et al., 2021).

Fungi and bacteria can both have intracellular and extracellular mechanisms of synthesis (Bhardwaj et al., 2020). Both mechanisms take place through different pathways in different microorganisms. In the intracellular synthesis mechanism, the transportation of positively charged metal ions takes place through the cell wall possessing a negative charge and are then internalized into the cells through various mechanisms, e.g., the synthesis of gold nanoparticles using *Lactobacillus kimchicus* (Mughal et al., 2021). In the extracellular synthesis mechanism, metal ions aggregate on the surface of a cell, e.g., the synthesis of gold nanoparticles using the *Paracoccus haeundaensis* bacterium (Cherian et al., 2022).

Similarly, fungi also synthesize nanoparticles by two pathways, but they are more prone to the extracellular type of synthesis. For example, the synthesis of silver nanoparticles by *Candida glabrata* (Ghosh et al., 2021). Among the available processes for the synthesis of metallic and metal oxide nanoparticles, the use of plant extracts for large-scale production instead of bacteria, fungi, and other microorganisms is a comparatively convenient process. To produce nanoparticles, plants are highly considered due to their biodiversity and availability of phytochemicals (Singh et al., 2018; Patil and Chandrasekaran, 2020). These phytochemicals show seasonal variations in yield, and nanoparticles synthesized by this method show a polydisperse nature. Phytofabrication is now considered a less time-consuming, safe, and cost-effective approach to constructing stable nanoparticles.

### 2.1 Plants

The plant is easily available and convenient to handle material compared to microorganisms in the biogenic synthesis of nanoparticles with efficient scale-up possibilities (Vijayaraghavan and Ashokkumar, 2017). The various constituents in plants, such as tannins, vitamins, alkaloids, polysaccharides, flavonoids, tannins, terpenoids, and saponins, play an important role in the bioreduction process for the synthesis of nanoparticles. The plant constituents also impart stability to nanoparticles (Patil and Chandrasekaran, 2020). Nanoparticle synthesis from plant biomass/extract points toward reducing generated waste and implementation of sustainability to the synthesized nanoparticles.

Various secondary metabolites, such as flavonoids, amino acids, phenolic compounds, enzymes, alkaloids, and glycosides, work as activators of the bioreduction of metal ions (BhardwajYadav et al., 2020). The advantage of using a plant is that every part consists of these metabolites; therefore, fruit, root, leaves, and bark can be used (Alamgir and Alamgir, 2017). Different constituents of extracts of blueberry, turmeric, and pomegranate when used for nanoparticle synthesis produced nanoparticles that have shown use in antioxidant therapy and cancer management as well (Gour and Jain, 2019). Many plants have applications in the synthesis of gold and silver nanoparticles, and most of them are medicinal plants such as Aloe vera, Alfalfa (*Medicago sativa*), Tulsi (*Ocimum sanctum*), Neem (*Azadirachta indica*), and Lemon grass (*Cymbopogon flexuosus*) (Singh et al., 2018). Extracts of leaves and fruits of Aloe vera, *Mangifera indica*, and Eucalyptus, bark extracts of *Boswellia ovalifoliolata and Cinnamomum zeylanicum*, and seed extracts of *Jatropha curcas* can reduce metals such as silver, gold, platinum, copper, cadmium, iron and zinc (Soni et al., 2021). Leaf extracts of *Magnolia Kobus* were found to be useful for the successful synthesis of copper and copper oxide nanoparticles (Lee et al., 2013). Aqueous extracts of *Gloriosa superba* and *Prosopis fractal* were used for the synthesis and stabilization of cerium oxide nanoparticles, which have applications in the treatment of obesity, and they were also used to immobilize cholesterol oxidase and glucose oxidase (Rocca et al., 2015). The generation of the first platinum nanoparticles was confirmed by *Diospyros kaki* leaf extract. Different phytochemicals present in plant extracts act as functional groups for reducing metals. The most common are flavonoids, which have lower molecular masses and are present in all parts of plants. The presence of flavonoids is an important factor in the use of plants for biogenic synthesis. Flavonoids decrease toxicity and stabilize nanoparticles (Shafey, 2020). Other significant phytochemicals participating in green synthesis are phenolic compounds, vitamins, and enzymes. Plants are nonpathogenic and the most suitable candidates in comparison to microbes for biogenic synthesis and can produce a variety of nanoparticles (Shobha et al., 2014).

### 2.2 Microorganisms

#### 2.2.1 Fungi

The use of fungi has some advantages compared to bacteria; fungi-mediated nanoparticle synthesis is environmentally friendly, the process is easy to scale up and economically viable, and the produced nanoparticles are very efficient, monodispersed, and have good morphologies (Kitching et al., 2015). Fungi contain enzymes that are more advantageous in biosynthesis than other microorganisms. The enzyme reductase in fungal cells carries out most of the metallic nanoparticle formation by the reduction process. Fungal cultures are preferred in the biogenic synthesis of nanoparticles due to their high biomass production. The handling of fungi is easier, and they show high tolerance toward various metals. Fungi secrete many proteins that impart stability to nanoparticles during biogenic synthesis. The fungal mass can easily tolerate high pressure and agitation and provides an advantage in scale-up. Adjustment of the culturing environment can execute controlled metabolism of fungi, which results in the synthesis of nanoparticles with desired characteristics (Guilger-Casagrande and de Lima, 2019).

Silver nanoparticles biosynthesized using fungi show wide applications as antimicrobial, antioxidant, and anticancer agents (Kaliammal et al., 2021). Multiple previous studies have emphasized the fungus-mediated synthesis of silver and other metal oxide nanoparticles. Few studies have demonstrated the use of *Cladosporium cladosporioides* in the synthesis of gold nanoparticles (Bhargava et al., 2016). *Fusarium oxysporum* fungus is widely used due to its potential for intracellular and extracellular synthesis of nanoparticles. Recent studies have shown the production of copper, magnesium oxide, gold, and platinum nanoparticles (Mukherjee et al., 2002).

Fungi are also capable of producing metallic oxide nanoparticles, and magnetite is a magnetic property containing iron oxide nanoparticles that can be produced by a fungus such as *Aspergillus* (Khandel and Shahi, 2018). These nanoparticles have found a variety of applications, such as MRI and position sensing. Zinc oxide nanoparticles synthesized by *Aspergillus niger* have shown exceptional antibacterial properties (Kalpana et al., 2018). Similar to bacteria, the use of fungi in biosynthesis also has some drawbacks regarding safety, and fungi such as *Fusarium oxysporum* are harmful because they are pathogenic. Nevertheless, the use of several nonpathogenic fungi has added benefits to green synthesis.

#### 2.2.2 Bacteria

Bacteria are abundantly available in the environment, and they can adapt to different environmental conditions, which helps them in the production of reduced metal ions. The production ability of nanoparticles using bacteria can be increased effectively with changes in the environmental conditions of bacterial cultures, such as pH, oxygen concentration, and temperature (Bahrulolum et al., 2021). Changes in bacterial culture conditions result in the formation of nanoparticles of different sizes. Bacteria are known for their ability to produce many unique nanostructures, such as nanomaterials and magnetic and metal oxide nanosystems. In particular, magnetotactic bacteria are adapted to build magnetosomes, which are nanocrystals of iron oxide and iron sulfide that are suitable for the production of magnetic radicals (Yan et al., 2017). These are applicable in molecular imaging, biosensors, and cancer therapy. Multiple bacteria have promising potential in metal ion reduction. The nanoparticles synthesized using bacteria show better stability and less agglomeration. The proteins obtained from bacteria act as capping agents and improve stability. Extremophilic bacteria can survive under extreme conditions and produce high-end nanoparticles (Atalah et al., 2022).

Many bacteria are known for the synthesis of silver nanoparticles with varying shapes and sizes, such as *Lactobacillus casei*, *Bacillus cereus, Arthrobacter gangotriensis*, *Pseudomonas proteolytic*, and many others (Thakkar et al., 2010). Similarly, several bacterial species known for the bioreduction of gold nanoparticles, such as *Pseudomonas aeruginosa* and *Rhodopseudomonas capsulate,* are broadly used (Singh and Kundu, 2014). Actinomycetes are a heterogeneous group of Gram-positive bacteria that have gained significant recognition due to their commercial necessity and ability for better intracellular production than any other biosynthesis contender. *Streptomyces*, Rhodococcus, and *Nocardia* have been identified for the synthesis of gold nanoparticles (El-Batal and Al Tamie, 2015). Cyanobacteria are the most abundantly present photosynthetic bacteria, and they primarily help stabilize nanoparticles. The cyanobacterium *Oscillatoria limnetica* has been successfully used in the synthesis of silver nanoparticles and further stabilization (Hamouda et al., 2019). There are certain limitations in the bacterial synthesis of nanosystems in purification and controlling the shape and size of produced monodispersed particles.

#### 2.2.3 Yeast

Yeasts are single-celled and eukaryotic types of fungi. Yeasts have also shown the conversion of toxic metal ions into nontoxic complex polymer compounds; these nanoparticles are present in the vicinity of yeasts, and they are referred to as “quantum semiconductor crystals” (Dameron et al., 1989). Yeast shows abundant secretion of extracellular enzymes responsible for the reduction of metal ions. The growth of multiple species of yeast is rapid, so it is easy to collect and preserve in the laboratory. The yield of nanoparticles obtained using yeast is large compared to bacteria, which is one of the prime advantages of using yeast as a source of biogenic nanoparticle synthesis (Boroumand Moghaddam et al., 2015). Several studies have demonstrated the synthesis of silver nanoparticles from *Pichia capsulata*, *Candida utilis* (Waghmare et al., 2015), *Rhodotorula glutinis* (Cunha et al., 2018)*,* and a silver-tolerant strain of the yeast *Saccharomyces cerevisiae*. *Hansenula anomala* can also be used as a catalyst in biofuels (Kalathil et al., 2013).

#### 2.2.4 Algae

Algae has an advantage in the synthesis of nanoparticles due to its significant potential for metal accumulation and metal reduction. The presence of bioactive compounds such as proteins in algae extract stabilizes the metallic nanoparticle, and they also act as a reductant. The live as well as dead biomass of algae is useful in the biogenic synthesis of nanoparticles. (Fawcett et al., 2017; Mukherjee et al., 2021). Algae has a rapid growth rate and high carbon dioxide sequestration, which makes it the candidate of choice for nanoparticle synthesis. Minimum energy inputs are required when algae are used for the fabrication of nanoparticles. One of the prime advantages of algae as a biogenic material for the synthesis of metallic nanoparticles is the generation of nontoxic byproducts. Multiple enzymes and pigments obtained from algae play important roles in bioreduction (Khan et al., 2022a).

Some brown algae secrete a polysaccharide called fucoidans, which is useful for synthesizing gold nanoparticles and provides an alternative to chemical methods. The use of *Sargassum muticum* was reported to decrease angiogenesis in HepG2 cells (Sanaeimehr et al., 2018). Various strains of algae, such as *Acanthophora spicifera*, *Laminaria japonica*, *Tetraselmis kochinensis*, and *Turbinaria conoides,* have potential applications for synthesizing gold nanoparticles (Senapati et al., 2012). Biosynthesis of nanocomposites from *Chlorella vulgaris* has shown bactericidal activity against multidrug-resistant *Staphylococcus aureus* (Kahzad and Salehzadeh, 2020). Algae are also an important green approach toward the synthesis of nanoparticles.

#### 2.2.5 Other sources

Microorganisms from marine sources are known for their tolerance to high salt concentrations and very extreme atmospheric pressure. Marine microorganisms reduce metallic ions and transform them into sulfides, carbonates, phosphates, or phytochelatins (Baker et al., 2013). Many metal nanoparticles have been produced using marine algae and marine plants. The synthesis of silver nanoparticles from brown seaweed *Padina tetrastromatica* showed promising antibacterial properties (Rajeshkumar et al., 2012). Similarly, regarding gold nanoparticles, brown seaweed (*Fucus vesiculosus*) showed gold nanoparticles of different sizes and morphologies (Mata et al., 2009). In addition to gold and silver nanoparticles, lead sulfide nanoparticles were also reported from a marine yeast, *Rhodosporidium diobovatum* (Seshadri et al., 2011)*.* Several nanoparticles synthesized from marine sources have shown a better and more promising advantage toward multidrug-resistant bacterial infections, which are a major global health risk. The variety and biodiversity in life forms in the marine environment along with the terrestrial environment guide researchers to utilize more sources in search of applications. Table 1 illustrates various sources for the biogenic synthesis of nanoparticles. The natural sources used for the synthesis of nanoparticles have advantages and disadvantages. The availability, efficiency, scale-up possibilities and safety are the parameters considered when selecting the biogenic source.

**TABLE 1:** Sources for biogenic synthesis of nanoparticles.

Sources	Nanoparticle type	Application	Intracellular/Extracellular synthesis	References
Bacteria				
*Escherichia coli*	Ag	Antimicrobial agent	Extracellular	Saeed et al. (2020)
*Cupriavidus* sp.	Ag	Antibacterial activity	Extracellular	Markus et al. (2016)
*Lactobacillus acidophillus*	Ag	Gene toxicity	Extracellular	Namasivayam et al. (2010)
*Lactobacillus kimchicus*	Au	Antioxidant	Intracellular	Markus et al. (2016)
*Paracoccus haeundaensis*	Au	Antioxidant	Extracellular	Patil et al. (2019b)
*Pseudomonas stutzeri*	Ag	Antimicrobial agent	Intracellular	Klaus et al. (1999)
*Staphylococcus aureus*	ZnO	Antibacterial	Intracellular	Rauf et al. (2017)
*Desulfovibrio vulgaris*	Platinum	Catalysts	Extracellular	Martins et al. (2017)
*Bacillus subtilis*	Fe_3_O_4_	Antimicrobial agent	Extracellular	Sundaram et al. (2012)
*Desulfovibrio vulgaris*	Palladium	Catalysts	Extracellular	Martins et al. (2017)
Fungi				
*Cladosporium cladosporioides*	Ag	Antioxidant	Extracellular	Zhang et al. (2020)
*Cladosporium cladosporioides*	Ag	Antimicrobial agent	Extracellular	Zhang et al. (2020)
*Aspergillus niger*	Ag	Antifungal agent	Extracellular	NevcihanGürsoy (2020)
*Cladosporium cladosporioides*	Au	Antioxidant	Extracellular	Bhargava et al. (2016)
*Cladosporium oxysporum*	Au	Catalysts	Extracellular	Bhargava et al. (2016)
*Fusarium oxysporum*	Cu	Antibacterial	Extracellular	Ghosh et al. (2021)
*Fusarium oxysporum*	Pt	Nanomedicine	Extracellular	Chatterjee et al. (2020)
*Aspergillus niger*	Cu	Antidiabetic	Extracellular	Zhang et al. (2019)
*Penicillium chrysogenum*	Pt	Cytotoxicity	Extracellular	NevcihanGürsoy (2020)
Yeasts				
*Candida guilliermondii*	Au	Antimicrobial agent	Extracellular	Mishra et al. (2011)
*Candida guilliermondii*	Au	Antibacterial	Intracellular	Agnihotri et al. (2009)
*Saccharomyces cerevisiae*	ZnS	Antibacterial	Intracellular	Sandana Mala and Rose (2014)
*Candida albicans*	CdS	Bactericidal	Intracellular	Patil et al. (2019b)
*Baker’s yeast*	Fe_2_O_3_	Detection of Glucose	Extracellular	Mishra et al. (2011)
Algae				
*Tetraselmis kochinensis*	Au	Antiviral	—	Senapati et al. (2012)
*Spirulina platensis*	Ag	Antiviral	Intracellular	Muthusamy et al. (2017)
*Spirulina platensis*	Ag	Antiviral	Extracellular	Muthusamy et al. (2017)
*Chlorella Vulgaris*	Pd	Adsorbent	Extracellular	Arsiya et al. (2017)
*Chaetomorpha linum*	Ag	Anticancer	—	Acharya et al. (2021)
Plants				
*Azadirachta indica*	Ag	Cytotoxicity	—	Potara et al. (2015)
*Artemisia annua*	Ag	Antibacterial	—	Khani et al. (2018)
*Brassica oleracea*	Au	Antimicrobial	—	Kumari et al. (2020)
*Tribulus terrestris*	Au	Antiulcer agent	—	Gopinath et al. (2019)
*Cassia occidentalis*	Cu	Hemolytic activity	—	Gondwal and Joshi nee Pant (2018)

## 3 Synthesis of biogenic nanoparticles

Two distinct fundamental synthesis principles (i.e., top-down and bottom-up) are used to fabricate nanomaterials with the required sizes, shapes, and functions (Biswas et al., 2012). Bottom-up methodologies have received significant interest in synthesizing biogenic nanoparticles. A variety of biological components, such as bacteria, algae, fungi, and plants, are useful for the synthesis of metallic nanoparticles (Begum et al., 2022).

**FIGURE 2:**
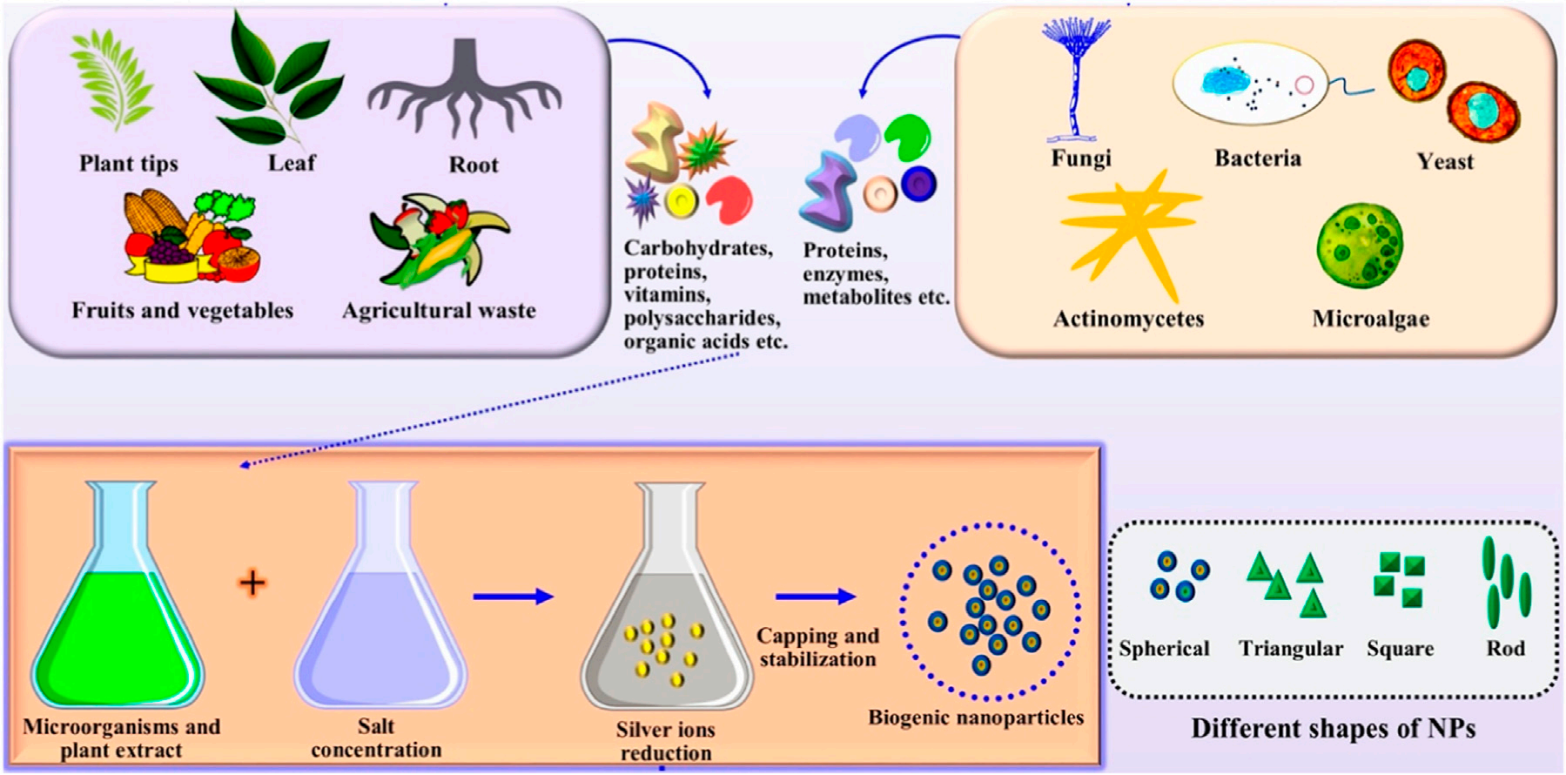
The synthesis of nanoparticles includes the extraction of various primary and secondary metabolites and subsequent reduction processes using appropriate ion concentrations. Depending on the process parameters used, the produced particles may have different shapes. Adapted from Ref (AliAhmed et al., 2020). CC BY license.

The solution of metal ions and a reducing biological agent are the two essential components needed for biogenic nanoparticles to be synthesized in a green manner. It is rarely necessary to introduce stabilizing and capping agents from outside because reducing agents or other components are by default available in the cells (Pal et al., 2019). Various methods of producing NPs utilizing plants and microorganisms have been reported in a variety of studies (Figure 2). Although there are many different green synthesis techniques for NPs, most of them depend on reacting organisms and biological reducing agents for the synthesis of metallic nanoparticles.

### 3.1 Plant-mediated biogenic synthesis of nanoparticles

In addition to its reduction ability, the plant extract acts as a capping and stabilizing agent. Plant-based NPs are suitable for photothermal therapy, biosensing, antioxidants, antimicrobials, and drug administration because they are biocompatible and exhibit distinctive chemical and optical properties (Begum et al., 2022). The advantages of plant materials in green synthesis are that they are nontoxic, inexpensive, readily available, and safe.

Bioreduction and biosorption are two important mechanisms for the synthesis of biogenic nanoparticles (Durán et al., 2011). Bioreduction is a method of chemically reducing metal ions to more stable forms via biological processes. This produces ineffective metallic nanoparticles as a result. It could be recovered from a compromised sample safely. In biosorption, the metal ions adhere to the biological organism, either from an aqueous sample or a soil sample. Some plants produce metal ions that bind to the cell wall and interact with a mixture of fungi, bacteria, and artificial peptides, creating a long-lasting nanoparticle structure (NadafJadhav et al., 2022; Sidhu et al., 2022). Khan et al. (2014) demonstrated the fabrication of palladium nanoparticles from *P. glutionsa*. In this process of synthesis, the reaction mixture was created by mixing palladium (II) chloride (PdCl_2_) solution with *P. glutionsa* plant extracts after stirring with a magnetic stirrer, and the pale-yellow color of the solution turned dark brown. There was no additional change observed even after stirring the mixture at 90°C for 2 h. The mixture was allowed to cool for 2 h before centrifugation (9,000 rpm). A black powder was formed after rinsing with distilled water, which was then dried overnight at 80 °C in the oven to obtain nanoparticles. The nanoparticles obtained from experimentation showed a particle size of 20–25 nm and were characterized by high-resolution transmission electron microscopy (TEM). FTIR analysis demonstrated the presence of various phytomolecules that act as capping agents, imparting stability to nanoparticles (Khan et al., 2014).

**FIGURE 3:**
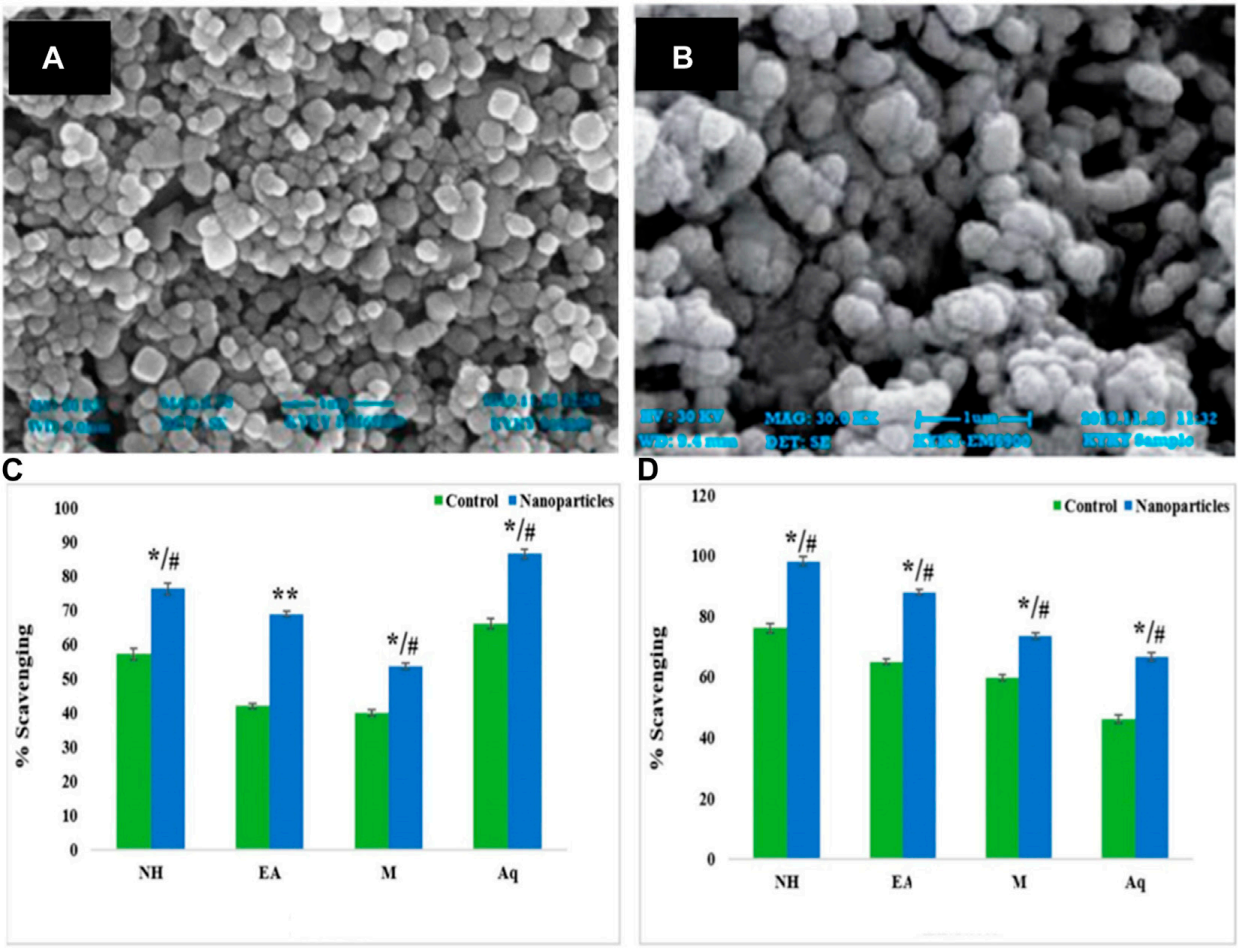
SEM images of biogenic ZnO NPs in the presence of aqueous extracts of leaves **(A)** and fruits **(B)**. The comparative DPPH scavenging antioxidant activity of control and ZnO NPs from **(C)** leaf and **(D)** fruit extracts of *C. colocynthis*. The mean ± SD is expressed as */#*p* < 0.05, **p < 0.01. Modified from (Kiani et al., 2023) CC BY license.

Kiani et al., (2023) synthesized ZnO NPs using leaf and fruit extracts of *Citrullus colocynthis*. These biogenic NPs resulted in a spherical surface morphology with a size range between 64 and 82 nm, as confirmed by SEM analysis (Figure 3). The 2,2-diphenylpicrylhydrazyl (DPPH) scavenging antioxidant study confirmed the highest activity in the aqueous extract compared to the n-hexane, ethyl acetate, and methanol extracts. In another study, Barzinjy *et al.* demonstrated the synthesis of zinc oxide nanoparticles from *Punica granatum* (pomegranate) juice extract (Alnehia et al. 2022; Barzinjy et al. 2020). The important chemical constituent present in pomegranate is polyphenol. A seed pulverization technique was used to prepare the pomegranate juice, which was further mixed with zinc nitrate (Zn (NO_3_)_2_) at 80 °C under controlled pH conditions with the gradual addition of sodium hydroxide (NaOH). The interaction between plant phytochemicals and metal salts results in a change in the color of the reaction mixture, which is an indication of nanostructure synthesis brought on by SPR’s appearance indicators compared to UV‒vis spectroscopy. In this study, the pink-colored solution changed to white, which shows the formation of zinc oxide (ZnO) NPs. The powder was obtained after centrifugation and cleaned using deionized water and methanol. The nanoparticles obtained by Barzinjy and coworkers show a particle size of 55 nm in SEM analysis. The synthesized nanoparticles were further subjected to the fabrication of a thin film. The fabricated film was found to be efficient with better crystallinity (Alnehia et al. 2022; Barzinjy et al. 2020).

### 3.2 Microorganism-mediated biogenic synthesis of nanoparticles

**FIGURE 4:**
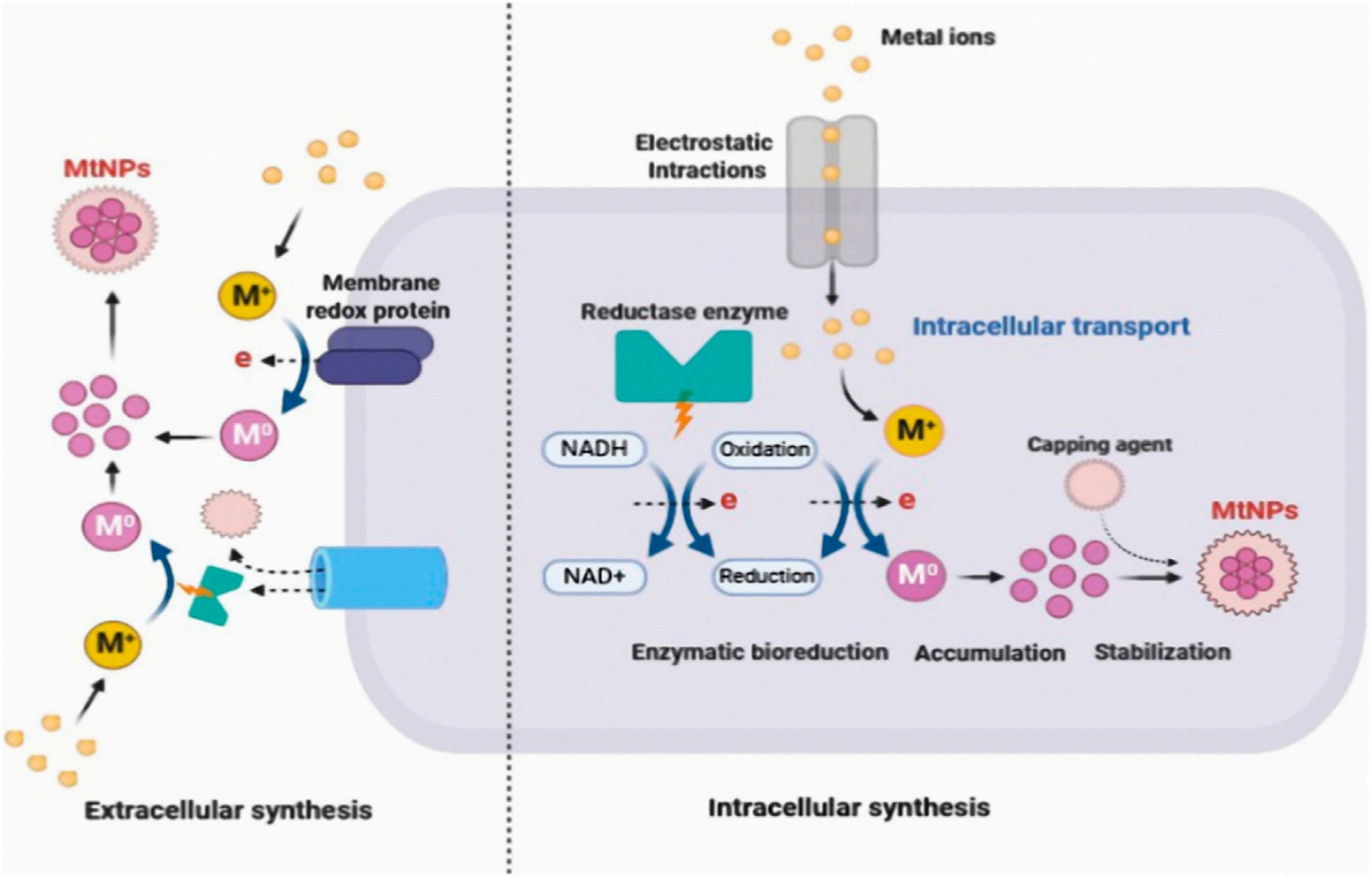
Mechanism of extracellular and intracellular biogenic synthesis of metal nanoparticles Adapted from Ref (Bahrulolum et al., 2021). CC BY license.

Microorganisms are important biofactories for the biogenic synthesis of metallic nanoparticles. This area has received significant attention because of its necessity and technological significance (Płaza et al., 2014). A wide variety of microorganisms react with metal ions to produce metallic nanoparticles in multiple ways. There are two different pathways for the synthesis of biogenic nanoparticles: intracellular and extracellular mechanisms (Figure 4). To counter various challenges, bacteria are developing a variety of defense mechanisms, such as internal sequestration, pumping efflux and modifying the metal ion concentration and extracellular precipitation (Naik and Dubey, 2013). These bacterial methods can be used in the environmentally friendly synthesis of nanoparticles.

Extracellular biosynthesis involves trapping metal ions on the cell wall and reduction in the presence of enzymes. Intracellular biosynthesis involves the transfer of metal ions into the cell cytoplasm and subsequent reduction.

Bacterial and fungal cells, as well as sugar molecules, play a vital role in the intracellular mechanism of metal bioreduction. Metal ions are primarily taken up by intracellular enzymes through interactions with positively charged groups, which leads to a decrease inside the cell (Ovais et al., 2018). The biomass is cultured with microorganisms under optimal growth conditions and then incubated with a metal ion solution to facilitate intracellular production. The color change indicates the synthesis of NPs. Furthermore, using ultrasonication, centrifugation, and washing, NPs are collected (AliAhmed et al., 2020). It is well known that microbial enzymes found extracellularly play an important role as reducing agents in the synthesis of metallic nanoparticles (Ovais et al., 2018). According to studies, cofactors such as nicotinamide adenine dinucleotide (NADH) and the reduced form nicotinamide adenine dinucleotide phosphate (NADPH)-driven enzymes have potential as reducing agents through the transfer of electrons from NADH (Guedes da Silva LeonorOlavarria Gamez et al., 2020).

The centrifuged culture filtrate is combined with an aqueous metallic salt solution for extracellular production (Luangpipat et al., 2011). The change in color of the combined solution is used to monitor NP synthesis. For instance, the color ranges from bright yellow to dark brown, indicating the production of silver nanoparticles (Jalab et al., 2021). *Streptomyces griseoruber* obtained from soil was cultured, and the supernatant was used to synthesize gold (Au) NPs extracellularly.

#### 3.2.1 Fungi-mediated biogenic synthesis of nanoparticles

Fungi are one of the prime biogenic sources for the synthesis of silver nanoparticles due to significant protein formation, high yield, and less toxic residues (Roy et al., 2019). Fungi play a key role in the reduction and stabilization of nanoparticles. Fungi are useful for the synthesis of extracellular and intracellular biogenic nanoparticles (Siddiqi and Husen, 2016). For intracellular synthesis, mycelial culture is incorporated with metal precursors and absorbed in the biomass. After synthesis, the nanoparticles are extracted by centrifugation, filtration, or chemical processing of biomass. In extracellular synthesis, the metal precursor is incorporated into the aqueous filtrate, which possesses fungal biomolecules in extracellular synthesis (Guilger-Casagrande and de Lima, 2019).

Dias *et al.* demonstrated the synthesis of zinc oxide nanoparticles from *Cordyceps militaris* (mushroom fungus). The obtained nanoparticles were characterized by FTIR, SEM, and XRD analysis along with polydispersity index, and the results were satisfactory. The particle size analysis demonstrated that the particle size was 1.83 nm with a PDI of 0.29, which shows the synthesis of quality nanoparticles. The zeta potential of −6.42 mV is an indication of the moderate stability of the nanoparticles. Biological evaluation revealed antidiabetic, antibacterial, and antioxidant potential (DiasAyyanar et al., 2022). Ameen et al., (2022) reported the synthesis of gold nanoparticles from *Alternaria chlamydospora* (marine fungus). The characterization supported the optimized synthesis of nanoparticles. This was the first study of gold nanoparticle synthesis performed using *Alternaria chlamydospora* at the international level. Spherical nanoparticles were obtained and confirmed with SEM. The synthesized nanoparticles exhibited antibacterial, anticancer, and antioxidant activity (Ameen et al., 2022). Bagur and coworkers synthesized silver nanoparticles from the endophytic extract of *Tinospora cordifolia*. The characterization and evaluation showed the successful synthesis of nanoparticles. The SEM and TEM analysis demonstrated a particle size of 25–35 nm with a spherical shape. DLS was performed to determine the hydrodynamic diameter, which was 65.2 nm. The zeta potential of −32.1 mV demonstrates the high stability of the nanoparticles due to abundant capping. The synthesized nanoparticles demonstrated antioxidant, antibacterial, and anti-inflammatory potential. The synthesized nanoparticles also showed antiproliferative potential against breast and cervical cancer cells (Bagur et al., 2022). Fungi are known to produce a variety of enzymes and metabolites that can act as reducing agents and stabilize nanoparticles. In addition, fungal-derived nanoparticles exhibit unique properties, such as increased stability, enhanced biocompatibility, biological activity, and low toxicity of the residues (Guilger-Casagrande and de Lima, 2019).

#### 3.2.2 Bacteria-mediated biogenic synthesis of nanoparticles

Bacteria are a promising choice for biogenic nanoparticles. Additionally, bacteria are capable of surviving in a variety of adverse circumstances, including extremes of alkalinity or acidity, high salt concentrations, and high- or low-temperature peaks. Because such compounds can be extracted from cells through cell filtration, which is thought to be advantageous, bacteria’s capacity to precipitate those chemicals out of cells makes it easier for them to produce nanoparticles biologically. The production of silver nanoparticles (AgNPs) by five different *Bacillus* strains was investigated, and only *Bacillus subtilis* has shown a stronger capacity for the production of these various compounds among these distinct *Bacillus* species AgNP (Alsamhary, 2020). The cell wall is used in intracellular synthesis to transport metal ions, where the positively charged ions interact with the negatively charged cell wall. These ions are converted to metal NPs in the cells by enzymes. Using the bacterium *Lactobacillus kimchicus* to synthesize gold nanoparticles, the process begins with the nucleation of chloroauric acid (HAuCl) ions, which results in the formation of nanoclusters via electrostatic interactions. Nanoclusters are then progressively transported across the bacterial cell wall. Metal ion accumulation on the surface of the cell and the involvement of reducing ions via enzymes are two aspects of the synthesis of nanoparticles extracellularly (Mughal et al., 2021).

The various isolates under examination were cultivated aerobically. On an orbital shaker, the microbial cultures were incubated at 37 °C with constant 200 rpm agitation. After 24 h of growth, the microbial biomass was collected, and centrifugation was performed at 10,000 rpm for 10 min. For the preparation of AgNPs, two solutions were prepared first, and some mL of supernatant was combined with 1 mL of silver nitrate (AgNO_3_) solution (1 mM) for the synthesis of AgNPs. The second reaction combination, which served as the control test, was made without AgNO_3_. For 24 h, the proposed solutions were incubated at 30 °C. To prevent any photochemical reversal throughout the experiment, all solutions were kept in the dark. The solutions then changed color from yellow to brown. The silver nanoparticles were collected for characterization after being purified by centrifugation twice for 5 min at 10,000 rpm. By measuring the optical density of silver nanoparticles produced by various cell supernatants, the most effective *Bacillus* strains for microbiologically synthesizing silver were identified. Within 18 h of incubation, the aqueous silver ions (Ag^+^) were converted to AgNPs when introduced to the cell-free supernatant of *B. subtilis*. The control showed no color change over the incubation time, whereas the yellow color turned brown (Kabeerdass et al., 2021). The bacterial synthesis of nanoparticles has limitations. The purification of nanoparticles and difficulty in controlling geometry as a result of a partial understanding of mechanisms are the key problems (Mughal et al., 2021).

#### 3.2.3 Virus-mediated biogenic synthesis of nanoparticles

Interest in the synthesis of nanomaterials by utilizing a virus as the aid template is growing. Due to their nanosize (20–500 nm), viruses are considered self-sufficient nanoparticles. Plant virology is the most explored subject to study the possible uses of plant viruses in nanotechnology. Plant viruses show no harmful effects on humans; therefore, they are most exploited for the fabrication of nanoparticles. The virus-mediated synthesis of nanocrystals has been studied by former researchers. The capsid protein present on the outer side of the virus plays a vital role in the biogenic synthesis of nanoparticles. The capsid protein provides a binding surface for metal ions with high reactivity (PanditRoy et al., 2022). Animal viruses also show a capacity for nanoparticle synthesis, but they are not studied very much due to safety concerns. The specific properties of viruses, such as their ability to produce nanoparticles in greater quantities, their biosimilarity, and easy gene manipulation for required properties, make them favorable agents for green chemistry-mediated synthesis (Mandhata et al., 2022). Bacteriophages are viruses that parasitize and reproduce only inside bacterial cells; because of their easy handling in laboratories, they are also used to study molecular biology.

*Cowpea mosaic viruses* (CPMV) are plant viruses with icosahedral symmetry (30 nm diameter). Due to their noninfectious nature toward mammals, they are used for nanoparticle synthesis. The absence of outer envelopes on the structure makes them perfect for nanomaterial production because the functionalities present on the capsid proteins are prone to direct contact with the coating material. The capsid of CPMV contains approximately 60 copies of both small and large proteins, and two ss-RNAs are also present in the same cavity (Sainsbury et al., 2010).

CPMVs are compatible with organic solvents and feasible for many hours at approximately 60 °C (Steinmetz and Evans, 2007). *In situ* vaccination of these viruses has shown a potent response against tumors in mouse models of glioma melanoma, ovarian cancer, and colon cancer. CPMV-Gold nanoparticles have shown potential applications in the immunotherapy of ovarian cancer (Kerstetter-Fogle et al., 2019). They have shown good efficacy against ovarian tumors (Gautam et al., 2021). *Tobacco mosaic virus* (TMV) has been used as a model for understanding the characteristics of viruses for over a century. It contains ss-RNA, which is present inside an assembly of capsid proteins. This assembly is tubular and is produced by the joining of 2,130 capsid proteins in a right-handed helical structure. Nanoparticles produced via TMV-mediated synthesis carrying cisplatin (an antineoplastic agent) have shown promise in the treatment of ovarian cancer (Franke et al., 2018).

M13 phage is a filament-like bacterial virus consisting of circular ss-DNA (Yacoby et al., 2006). Bacteriophages are viruses that have infected bacteria, due to which they can be easily multiplied in laboratories using *E. coli*. In the synthesis of barium titanate nanoparticles, M13 phages were the only support used (Jeong et al., 2013). The M13-Magnetic nanoparticles exhibit peptide targeting in prostate cancer, which is of very importance in Magnetic Resonance Imaging (MRI). Bacteriophage nanoparticles combined with chloramphenicol have shown promising action against *Staphylococcus aureus* (Yacoby et al., 2006).

#### 3.2.4 Yeast-mediated biogenic synthesis of nanoparticles

**FIGURE 5:**
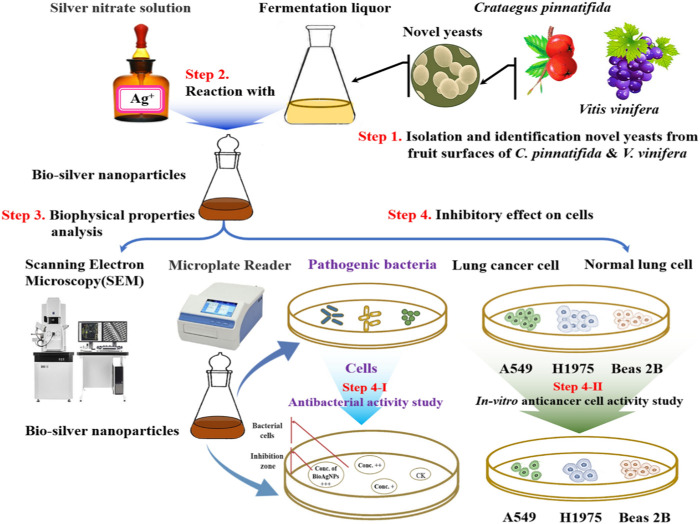
Experimental diagram of the synthesis of Ag nanoparticles from the yeast strain. Adapted from (Liu et al. 2021) CC BY license. Various steps include isolation of two yeast strains, HX-YS and LPP-12Y, and analysis of their activity on the human lung cancer cell lines A549 and H1975, as well as the normal human lung epithelial cell line Beas 2B.

Yeast has additional advantages over bacteria due to the high yield of NPs as well as the ease of manipulating yeasts in available laboratory settings, the synthesis of multiple enzymes, and the quick development using simple nutrients (Süntar et al., 2021). The synthesis has been the subject of some investigations used to create metallic nanoparticles using yeast. The synthesis of silver nanoparticles from yeast extract is reported in this study. *Saccharomyces cerevisiae* powder was dissolved in deionized water to prepare a solution (Roy et al., 2015). The silver nitrate solution was also prepared in deionized water. The yeast extract solution (*Saccharomyces cerevisiae*) was used to decrease the silver ions in the AgNO_3_ solution to create silver nanoparticles. With exposure to the fungal extracts, the yeast extract solution was added dropwise to the AgNO_3_ solution, which resulted in the formation of silver nanoparticles, which were visually observed by the change in color (Liu et al., 2021). Yeast strains of HX-YS and LPP-12Y were also found to be useful in the synthesis of biogenic nanoparticles. The strains HX-YS and LPP-12Y were isolated from *Crataeguspinnatifida* and *Vitis vinifera*. The addition of silver nitrate solution to the yeast extract resulted in the synthesis of biogenic nanoparticles by bioreduction. Centrifugation was performed to obtain the nanoparticles from biomass (Figure 5) (Liu et al., 2021).

Salem (2022) demonstrated the use of baker’s yeast for the synthesis of selenium nanoparticles. *Saccharomyces cerevisiae* (baker’s yeast) showed promising results in this green synthesis, and the resulting nanoparticles had potential antimicrobial activity against pathogens.

#### 3.2.5 Algae-mediated biogenic synthesis of nanoparticles

The development of nature-friendly techniques has been sparked using several species of algae for synthesizing metallic NPs (Lateef et al., 2016a). The synthesis of biogenic nanoparticles utilizing algae as a biological template or reducing agent is known as algae-mediated synthesis. The process can be carried out using various algal species, including green algae, diatoms, and cyanobacteria. The synthesis of biogenic nanoparticles from algae typically involves the addition of a metal salt solution to an aqueous suspension of algae, followed by incubation under specific conditions (Mishra et al., 2020; Sinha et al., 2015). Algae-mediated silver NPs are gaining much attention because of their powerful antibacterial properties. As a result, synthesis occurs when microalgae are present, and the metabolites that the algal culture excretes lead to a decrease in silver ions (Merin et al., 2010). Algae usage is beneficial for the synthesis of nanoparticles because they have a negative charge on the surface of the cell, which leads to rapid nucleation and crystal formation, and they are extremely affordable for large-scale synthesis (Jena et al., 2014). It was discovered that the synthesis of calcium carbonate (CaCO_3_) took place due to the charged surface of microalgae cells. The positively charged Ca^2+^ ions aggregated on the surface of negatively charged algae cells and resulted in the start of the nucleation process. The level of Ca^2+^ ions also had a significant impact on the dimensions of the microspheres, and the CaCO_3_ crystal size increased with concentration. Keeping the number of algal cells stable and promoting heterogeneous nucleation. The obtained microspheres were found to be useful for the synthesis of Ag NPs (Sahoo et al., 2014).

According to reported research, ZnO NP synthesis was carried out by combining dried powder of *S. muticum* algae with distilled water and heating it until it was well mixed (Uzair et al. 2020). By adding zinc acetate salt solution and then stirring the mixture for hours, NPs were produced. The synthesized ZnO NPs displayed a hexagonal shape, had bioactive functional groups such as sulfate, amine, hydroxyl, and carbonyl groups, and ranged in size from 35 to 57 nm (Chaudhary et al. 2020). Bharathi et al. (2022) demonstrated the applications of brown seaweed algae in the synthesis of silicon dioxide (SiO_2_)–ZnO nanocomposites. *Dictyota bartayresiana* (brown algae) extract was used in this green synthesis. The resulting nanoparticles demonstrated potential anticancer, antibacterial and antioxidant activity.

The green nanoparticles synthesized from algae also showed antiviral potential. The study reported the biogenic synthesis of silver and gold nanoparticles. Blue‒green algae, namely, *Oscillatoria sp*. and *Spirulina platensis* were used for synthesizing these nanoparticles (Mandhata et al., 2022). The proteins and polysaccharides in algae exhibit bioreduction, stabilization, and capping in the synthesis of nanoparticles. These biogenic nanoparticles were found to be useful against herpes simplex virus (El-Sheekh et al., 2022).

### 3.3 Enzyme- and vitamin-mediated biogenic synthesis of nanoparticles

Biomolecules such as proteins, enzymes, and polysaccharides are frequently used in the biological synthesis of nanoparticles. Enzymes have very distinctive structures, and they can catalyze reactions without interfering. Enzymes show reducing and stabilizing effects on nanoparticle synthesis. The biogenic nanoparticles obtained using enzymes show high stability and less agglomeration (Ovais et al., 2018). Gold nanoparticles synthesized by using keratinase enzyme produced by bacteria inhibit *E. coli* and S*. aureus*. Similarly, the synthesis of AgNPs with antifungal activity was developed by sulfite reductase enzymes (Gholami-Shabani et al., 2015). Sensor applications of silver nanoparticles were also detected, and they were synthesized by redox enzymes. These nanoparticles worked as the electron-transmitting agent between electrodes and the biocatalyst.

Vitamin B2 showed capping and reducing properties in nanoparticle production. A combination of chitosan and vitamin C (ascorbic acid) can be utilized for the fabrication of nanostructures, and the ability of chitosan to form chelates has encouraged the mass production of ‘chitosan-metal complexed nanoparticles’. The H^+^ ions in plants are accompanied by NAD, and they show promising reducing activity, which can be favorable in the synthesis of gold nanoparticles (NadafJadhav et al., 2022). The stability of biogenic nanoparticles is affected by factors such as pH, temperature, and ionic strength, which can cause the capping agents to detach from the nanoparticle surface and lead to aggregation (Patil and Chandrasekaran, 2020).

### 3.4 Gum, resin, and molasses-mediated biogenic synthesis of nanoparticles

Plant waste materials are being researched for their potent use in nanotechnology, which can be useful for nontoxic and biodegradable nanoparticle synthesis (Saratale et al., 2018; Chhabra et al., 2020). The different byproducts of plant metabolism can be applied for the synthesis of nanoparticles such as gums, resins, and biowaste of sugar crystallization “molasses.” Asafoetida and Acacia can be employed in the green synthesis of stable silver nanoparticles, which show applications in the treatment of resistant antimicrobial infections and periodontal diseases (Devanesan et al. 2020). Asafoetida is an herb derived from dried latex of the rhizome of ferula foetida. The main composition of this latex is resin and gum 60% and 25%, respectively. Silver nanoparticles blended with asafoetida powder result in a therapeutic complex of nanoparticles and asafoetida, which can be very useful to stop the growth of both Gram-negative and Gram-positive bacteria. They also found their effectiveness in the treatment of periodontal disease (Devanesan et al. 2020). Acacia gum can also be incorporated in the stabilization of silver nanoparticles, and these nanostructures have found applications against *Candida* albicans (Alqarni et al. 2022). Molasses is a byproduct acquired from the crystallization stage in the sugar cane refinery. Molasses is the thick, viscous, and dark brown secondary waste in sugar refineries. Abundant polyphenol content is liable for antioxidant properties. These unwanted materials can be used in silver nanoparticle synthesis. It is a very cost-efficient method for the preparation of silver nanoparticles. There are abundant sensor applications for these fabricated bio nanoparticles, mainly to detect the concentrations of metallic ions in the environment (Vonnie et al. 2022). The biogenic synthesis of nanoparticles is based on the reaction between biogenic and metallic precursors. Bioreduction is an important reaction that takes place during synthesis. Intracellular and extracellular mechanisms depend on the type of biogenic source for synthesis. The biochemicals acting as capping agents impart stability to the synthesized nanoparticles.

## 4 2D biogenic nanoparticles

Biogenic 2D nanomaterials are produced using natural sources that are readily available and eco-friendly. The synthesis process is less toxic, less expensive, and less energy-intensive than synthetic methods. Biogenic 2D nanomaterials are inherently biocompatible, making them suitable for biomedical applications. Plants can be used to synthesize a wide range of 2D nanomaterials, including graphene, graphene oxide, and metal oxide nanoparticles. The process involves the use of plant extracts as reducing agents and stabilizers, which can be obtained through simple extraction methods (Ullah and Lim, 2022). Bacterial microorganisms can be used as an aid to produce 2D nanomaterials, including graphene oxide, by exploiting their potential to reduce metal ions. Fungi can also be used to synthesize nanomaterials by using their extracellular enzymes to reduce metal ions (Rai et al., 2023). Other materials used for synthesis are rice husk leftover, tea tree extract, waste food, onion sheathing, dead leaves, *etc.*

One of the most popular ways to prepare nanomaterials is through hydrothermal synthesis. It is simply a solution reaction-based method. Hydrothermal synthesis is a method that involves the use of high temperature and pressure conditions to promote the synthesis of 2D nanomaterials. The process involves the use of natural precursors such as plant extracts, which act as reducing agents and stabilizers. The synthesis is typically carried out in a high-pressure reactor, and the reaction conditions are optimized to promote the growth of the desired 2D nanomaterial. Some examples of hydrothermally synthesized 2D nanomaterials include carbon quantum dots (CQDs), ZnO-ZrO2 nanocomposites, and nickel ferrite (NiFe_2_O_4_) nanoparticles (Gan et al., 2020). For instance, the synthesis of chitosan-MoS2 hybrid nanocomposites involves the dissolution of chitosan in acetic acid solution and the addition of MoS_2_ nanosheets followed by sonication and centrifugation to obtain chitosan-MoS_2_ hybrid nanocomposites (Kasinathan et al., 2020).

2D nanomaterials and current developments are influencing biomedical science through various applications (Marian et al., 2022). With their capacity to adsorb several drug molecules and give users more control over release kinetics, 2D nanomaterials are being investigated for application in drug delivery systems. They are also helpful for enhancing the mechanical characteristics of biomedical nanocomposites, even at low concentrations, due to their extraordinary surface area-to-volume ratio and often high modulus values (Chimene et al., 2015). For multimodal imaging of tumors, 2D nanomaterials may potentially be created as nanoprobes. Chitosan, MoS_2,_ and their hybrid nanoconjugates show antibacterial activity against *Escherichia coli* and *Streptococcus* species. The inhibition rate of *S. aureus* was lower than that of the *E. coli* species. Similarly, the anticancer activity of the chitosan-MoS_2_ nanomaterial was investigated. The green synthesized chitosan and MoS_2_ composites were minimally toxic with high biocompatibility (Fan et al., 2015). The anticancer property was evaluated against breast cancer cells (MCF-7), and the evaluation resulted in the finding that the activity was dose-dependent (Lu et al., 2017). Metal nanoparticles are frequently used on 2D platforms to attach antibodies and improve signal quality for electrochemical biomarker detection (Koyappayil et al., 2023).

## 5 Characterization of biogenic nanoparticles

Nanoparticles have characteristics that make them valuable in a wide range of applications. For biomedical applications in particular, it is essential to characterize them thoroughly to determine whether they are suitable for the intended use. This is accomplished by utilizing several techniques and equipment that can provide the required information (Aljeldah et al., 2023).

Field emission scanning electron microscopy (FESEM) is a microscope that employs negatively charged electrons opposite of light. These electrons can scan things in a zig-zag manner. An emission source frees electrons propelled by a high electric field gradient (Ebrahimzadeh et al., 2023). Approximately all of the NPs in the FESEM micrograph should be perfect round spheres, which seems to be a frequently noticed characteristic of biosynthesized NPs (Nile et al., 2023).

UV visible microscopy is useful to determine surface plasmon resonance. The change in color of the reaction mixture from light yellow to brown was induced by the formation of AgNPs because of AgO reduction from Ag+ ions. The surface plasmon resonance absorption band is produced by mutual vibrations of free electrons of AgNPs concerning the metal lattice in resonance with the electromagnetic field of light waves, which oscillates (Judefeind et al., 2009).

Auger electron spectroscopy (AES) provides information about a nanoparticle by describing Auger electrons in atoms at various depths and provides elemental maps and depth profiles. Except when depth profiling necessitates surface scraping, it is often nondestructive (Singh, 2016). Secondary ion mass spectrometry (SIMS) is one of the most often used imaging techniques in desorption/ionization mass spectrometry. The SIMS approach is fundamentally surface sensitive, with information often coming from the first few atomic layers, equating to depth information of only a few nanometers (2–5 nm range) (Schaepe et al., 2020).

Fourier transform infrared spectroscopy (FT-IR) was also used to confirm the potential role of NP production. It determines the functional groups on the surface of nanoparticles by detecting chemical bond excitations. The molecular data obtained give structural and conformational changes. Wavenumbers showed the interaction between the capping agent and the NPs (Salem et al., 2022).

Powder XRD is a prominent approach to examining the physicochemical composition of unknown materials. XRD is a straightforward method for identifying the size and shape of a unit cell in any substance. This technique is useful for qualitative, quantitative, and other forms of analysis. Peak locations reflect translational symmetry, especially the unit cell’s size and form. On the other hand, peak intensities offer data on electron density within the unit cell, i.e., where the atoms are situated (Thakar et al., 2022). The XRD analysis confirmed the nature of the biologically generated NPs. The particular spectrum displayed peaks from XRD. The XRD peak patterns and different diffraction peak values, which show the reflection planes, demonstrate the nature of the produced materials (Oves et al., 2023).

Dynamic light scattering (DLS) analysis is an intensity-based method that places more focus on particles with a large particle size in mixed solutions. DLS analyses the hydration sphere diameter of the NP and is a gradual analysis approach that measures thousands of nanoparticles in the mixed solution (Das et al., 2023). Selected area electron diffraction (SAED) is a method that may be used with TEM to discriminate between crystalline and amorphous materials, and it can be used to validate the findings of XRD analysis (Mahakham et al., 2016).

The colloidal stability of the NPs is tested using zeta potential measurements, and it is thought that the results recorded over 30 mV in magnitude show that the nanoparticle sample is mostly stable in terms of colloidal stability (Alzubaidi et al., 2023). Using zeta Sizer, a high-performance molecular size analyzer, one may determine the stability and charge of nanoparticles at a pH of 7 by measuring their zeta potential (Pete et al., 2023).

The polydispersity index (PDI) is used to obtain a measure of the width of a molecular weight distribution. The size distribution of a particle sample directly corresponds to the PDI values. Monodisperse samples have PDI values below 0.05, while samples with a wide range of particle sizes are more likely to have PDI values above 0.7. The high negative charge of the biogenic NPs indicated that the surface of these nanoparticles was negatively charged (Aljeldah et al., 2023). Using Brookhaven Zeta Plus and dynamic light scattering (DLS) measurements, the hydrodynamic size of the phytofabricated NPs and PDI were assessed using nanoparticle-tracking analysis. The zeta potential was used to calculate the net surface charge (Ebrahimzadeh et al., 2023).

When nanoparticles are taken into account for drug delivery purposes, they are first checked for drug loading and entrapment efficiency. Drug loading is the procedure in which the drug is contained in the polymer matrix or the core of the nanoparticle. The amount of the drug that is incorporated and by the means it is included in the nanoparticle governs the performance of the drug release. Drug release is the opposite process of drug loading; in drug release, the entrapped drug becomes available for absorption after being released from the core. Therefore, the drug-carrier relationship can be influential in predicting the *in vivo* performance of the drug.

The drug is loaded into the nanoparticle core by different drug loading methods, such as hydrogen bonding, ionic bonding, covalent bonding, dipole interaction, and physical methods, such as encapsulation, or it can be adsorbed on the surface of the nanoparticle. There are two ways to entrap a drug: entrapment during the synthesis of nanoparticles and entrapment after synthesis. The interaction between the drug and carrier system is taken into consideration before drug loading, as it increases the entrapment efficiency and drug loading, but a decrease in the release rate can be seen (Bhattarai et al., 2006).

The entrapment efficiency of the nanoparticles is a variable used to estimate the percent drug loading. Entrapment efficiency is the method used to evaluate the total percentage of the drug that has been loaded into the nanoparticle. The higher the entrapment efficiency is, the greater the drug loading capacity (Judefeind et al., 2009). Table 2 illustrates the various general characterization techniques for nanoparticles.

$$Drug\;loading\;\left(\%\right)=\frac{Mass\;of\;drug\;in\;Nanoparticles}{Mass\;of\;Nanoparticles}\times 100$$

$$Entrapment\;efficiency\;\left(\%\right)=\frac{Experimental\;drug\;loading}{Nominal\;Drug\;loading}\times 100$$

**TABLE 2:** General characterization techniques for biogenic nanoparticles.

Sr. No.	Techniques	Principle	Purpose
1	UV‒Vis Spectroscopy (UV‒Vis)	Measurement of distinct spectra produced after the absorbance of the ultraviolet and visible light	Identification of compound and concentration determination
2	X-ray Diffraction (XRD)	Irradiation of X-rays on a sample and then measuring the scattering angle and intensity of X-ray	Crystal structure determination, Particle size determination
3	Differential Scanning Calorimetry (DSC)	Measure the change in physical properties of a substance, with change in temperature along with time	Measurement of change in glass transition temperature and melting point
4	Fourier Transform Infrared Spectroscopy (FTIR)	Covalent bonds of the sample absorb infrared radiation and the transmitted radiation is measured	Determination of functional groups and chemical composition
5	Transmission and Scanning Electron Microscopy (TEM and SEM)	Transmission and scanning using electrons and detection by detectors	To determine surface morphology of nanoparticles
6	Field Emission Scanning Electron Microscopy (FESEM)	Particles emitted by the field emission gun scan the sample object according to a zig-zag pattern	Determination of microstructures
7	Auger Electron Spectroscopy (AES)	Analyzing the element constituting the sample surface	Elemental and chemical characterization
8	Secondary Ion Mass Spectrometry (SIMS)	The primary ion beam spits the secondary ion from the sample surface and examination of secondary ions	Analyze the composition of surface
9	X-ray Photoelectron Spectroscopy (XPS)	Sample electrons absorb specific energy photons and are emitted and then kinetic energy analysis is done to study electronic states	Elemental as well as chemical composition
10	Dynamic Light Scattering (DLS)	Evaluating the light interference based on the Brownian motion of nanoparticles	Evaluate nanoparticle size
11	Zeta potential	Investigation of surface charge by simple attraction and repulsion study between counter and coions	Determination of surface charge

## 6 Biomedical applications

Nanotechnology involves the understanding and control of matter at the nanoscale. Their smaller size and unique qualities make them more useful in multidisciplinary applications in optoelectronics and photovoltaic, biomedical, and thermoelectric spinning (Roco et al., 2011). Biogenic nanomaterials have multiple advantages due to their friendly approach along with diverse biomedical applications, such as antimicrobial (Benko et al., 2023), biosensor, anti-filarial, antioxidant, and antileishmanial applications.

### 6.1 Antibacterial applications

**FIGURE 6:**
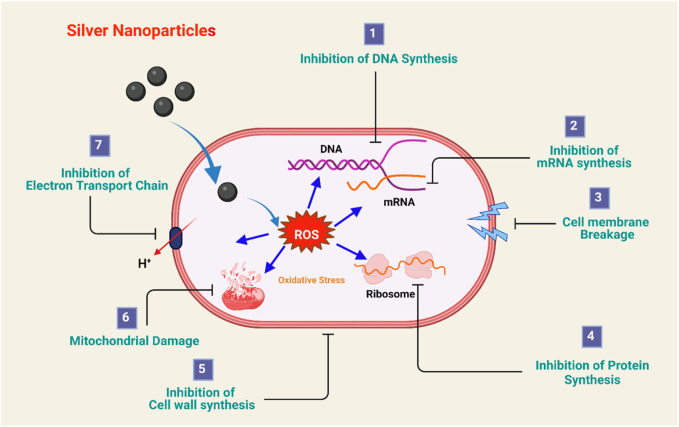
Antimicrobial mechanism of silver nanoparticles. Adapted from (Jain et al., 2021) CC BY license.

High-yield gold nanoparticles developed from the extraction of banana peels can be used in biomedical applications and against some harmful bacteria, such as *E. coli* (SI et al., 2020). Researchers have demonstrated the antibacterial applications of biogenic iron nanoparticles (BINPs) obtained from spinach leaf extract and banana peel extract against *E. coli and B. subtilis*. The bactericidal activity of banana peel extract-based iron nanoparticles (FeNPs) and spinach leaf extract-based SLE-FeNPs did not differ noticeably. Additionally, it has been shown that these NPs penetrate microbial membranes and result in electrolyte leakage, which eventually results in cell death. Nanomaterials can increase the permeability of bacterial cell membranes, prevent bacterial protein denaturation and DNA replication and release silver ions (see Figure 6 for various mechanisms) (Tyagi et al., 2021).

Antimicrobial properties are beneficial to silver nanoparticles. AgNPs (silver nanoparticles) were synthesized using a Daucus carota extract, and ceftriaxone was added to increase their antibacterial potency (Shanmuganathan et al., 2018; Talank et al., 2022). The antibacterial activity of biogenic AgNPs was evaluated using the good disc diffusion technique. The experiment was run against Gram-positive and Gram-negative bacteria as controls. The discrete, spherical-shaped AgNPs containing ceftriaxone-polydispersed aggregates displayed smooth, agglomeration-free surfaces (Shanmuganathan et al., 2018). To determine the potential antimicrobial use, cinnamon tamala leaf extract was employed. The MIC value was calculated. The biosynthesized AgNPs were tested for antimicrobial applications against multidrug-resistant *E. coli* and *Klebsiella pneumonia* as well as Gram-positive *S. aureus*. Treatment with AgNPs showed concentration-dependent bacteriostatic activity against all three bacterial strains. In this nanocomposite, several plants, phytochemicals, and proteins were useful as capping agents. A time-dependent reduction in bacterial population growth was achieved using AgNPs. It is also an effective inhibitor of biofilm formation (Dash et al., 2020). *Piper nigrum* (black paper) leaf extract is used as a reducing and stabilizing agent along with silver nitrate as a precursor in the biogenic synthesis of silver nanoparticles. The moderate particle size and shape of the generated nanoparticles depend on the molar content of the silver nitrate solution used. It is demonstrated that the generated nanoparticles have numerous amide-containing compounds. The evaluation showed the crystalline nature of the generated nanoparticles. The results of this study were equivalent to those investigations where chemical-reducing agents such as sodium citrate were used (Augustine et al., 2014). Silver nanoparticles from *cola nitida* showed antimicrobial effectiveness against *E*. *coli*, *P. aeruginosa*, *A. niger*, *A. fumigatus*, and *A. flavus*. The use of innovative nanomaterials, such as nanoparticulate silver, in the paint industry may profit from improving the quality of the paint with antimicrobial activity. This is indicated by the possible application of nanoparticles as additives in paints (Jain et al., 2021).

The synthesis of copper nanoparticles (40–100 nm) is reported from Magnolia leaf extract, which acts as a reducing agent. The resulting nanoparticles showed significant antibacterial activity against *E. coli.* Foam sprayed with biologically synthesized copper nanoparticles showed greater antibacterial activity than untreated foam (Rubilar et al., 2013). Nanoparticles show intrinsic toxicity to bacteria as a result of their high surface area to volume ratios, which makes their entry into bacterial cells easier and facilitates their interaction with functioning biomolecules such as DNA and proteins (Jacob et al., 2019). Fenugreek, or Trigonella foenum graecum (L.), is a fragrant leguminous plant. The silver nanoparticles produced by fenugreek seed extract have a significant antibacterial effect against *E. coli*. Notable antibacterial activity was also detected against *B. cereus and S. aureus*. It was found to be effective against both Gram-positive and Gram-negative bacteria, which is consistent with other results. When the nanoparticles are connected to the membrane of the cell, they interact with membrane proteins that contain sulfur to cause them to permeate the bacterial cell wall. This is similar to how bacteria interact with phosphorus-containing substances such as DNA, inhibiting DNA replication and cell reproduction (Awad et al. 2021).

### 6.2 Anti-malarial applications

Biogenic nanoparticles have significant applications in the prevention and treatment of malaria. Zornia diphylla aqueous extract proved harmful to the mosquito vector larvae Anopheles subpictus, Culex tritaeniorhynchus and *Aedes albopictus* (Ga’al et al., 2018; Govindarajan et al., 2016). The percentage of fatality was proportionate to the lethal concentration assessed, according to the toxicity data. AgNP produced from *Gmelina Asiatica* leaf extract was found to be effective against numerous mosquito vectors, with *Anopheles stephensi* showing the maximum larval mortality, followed by *Aedes aegypti* and *Culex Quinquefasciatus*. Silver nanoparticles derived from *Drypetes roxburghii* fruit extract exhibit some promising properties for mosquito biocontrol, particularly during the larval stage. The nanoparticles produced from putranjiva have a substantially higher lethal concentration value for *Anopheles stephensi*. The AgNPs pass through the larval membrane, causing the fragile larvae to die. The linearity between the dose of AgNPs and the mortality of larval mosquitoes shows the dose-dependent antimalarial potential and larvicidal activity. The study revealed the linear relationship between the concentration of silver nanoparticles and associated larvicidal action in the case of both mosquitos (*Aedes aegypti* and *Culex Quinquefasciatus*) (Haldar et al., 2013).

### 6.3 Anti-leishmanial applications

Leishmaniasis is a parasite disease produced by Leishmania species that is one of the most serious (Singh and Sivakumar, 2004). Green nanoparticle-based therapeutic approaches for cutaneous leishmaniasis are being developed to combat both promastigote and amastigote types (Shah and Gupta, 2019). The antileishmanial activities of *Cuminum cyminum*-based biogenic silver nanoparticles in macrophages were driven to create NO, which eliminates Leishmania parasites. Additionally, in addition to their ability to kill parasites directly, they have the power to cause immune cells to suppress parasites. Studies have demonstrated that metal oxide nanoparticles are quite successful in treating leishmaniasis. When assessing the infection index, *L. tropica* parasites were exposed to Bio-AgNPs at a dose of 1.75 g/mL, and their infectivity rates were reduced. The findings showed that whereas AgNPs caused a notable decrease in macrophage cell viability values at the same doses, Bio-AgNPs had no appreciable cytotoxicity toward them at lower concentrations (0.25–0.75 g/mL). The antileshminial activity observed is the result of inhibition of promastigote viability and disturbance in the metabolism of amastigotes along with enhanced release of nitric oxide from host macrophages (Bagirova et al., 2020).

### 6.4 Anti-filarial applications

*Culex quinquefasciatus* is a vector that spreads lymphatic filariasis. AgNPs have larvicidal action (Shanmugasundaram and Balagurunathan, 2015). Actinobacterial isolates have also been reported to have high larvicidal action against Anopheles mosquito larvae (Sutthanont et al., 2019). Actinomycetes also have significant efficiency against *C. quinquefasciatus* (Tanvir et al., 2014). Because of their distinctive shape-dependent chemical capabilities, these biological entities may be used to create nanoparticles, which is significant for nanobiotechnology. In these scenarios, a novel approach employing biologically generated AgNPs with considerable effectiveness against mosquito larvae was assessed. A substitute for current chemical larvicides is the biogenic nanoparticles produced by fungi and bacteria. Multiple bacterial and fungal isolates have been tested for their ability to produce silver nanoparticles in previous studies. The larvicidal activity of nanoparticles of biogenic origin has been evaluated in the past. The isolates of *A. bisporus, E. coli, Penicillium* sp.*, and Vibrio* sp. showed considerable larvicidal activity (Dhanasekaran and Thangaraj, 2013). The aqueous extracts of *L. aspera* were very vulnerable to the *L.* aspera AgNP concentration, with 100% mortality reported. The nanosized silver particles generated from the leaves of *L. aspera* and *H. suaveolens* were found to be very stable and have considerable mosquito larvicidal action against *Aedes aegypti*, *Aedes stephens*, and *C. quinquefasciatus* larvae. This demonstrates AgNP potency as a robust larvicidal agent. Capping enables surface reactivity, which makes these functionalized nanoparticles important against these vectors. In combination with mosquito-repellant formulations, these biogenic silver nanoparticles can have promising applications in the prevention and treatment of malaria and filariasis (Elumalai et al., 2017).

### 6.5 Dental applications

AgNPs are regarded as potential antibacterial agents for dental materials in dentistry, and their inclusion in resin materials is considered a viable option to increase the longevity of dental restorations (Bapat et al., 2018). Even at low doses, AgNPs demonstrate antibacterial activity against *Streptococcus mutans*, which causes dental caries. *Camellia sinensis* (green tea plant) extract was used to create AgNPs. *Camellia sinensis* contains a high concentration of polyphenolic chemicals, particularly catechin, which serves as a reducing and capping agent. A light-colored SiO_2_ coating was applied to the surfaces of AgNPs to enable their prospective use in dentistry (Rajan et al., 2015).

The red seaweeds *Solieria robusta* and *Halymenia porphyriformis* were found to be useful in the green synthesis of silver nanoparticles (Khan et al., 2022b). These nanoparticles from biogenic origin were investigated for their ability to inhibit oral pathogenic microorganisms that cause cavities or tooth decay. Smaller particles have a higher surface-to-volume ratio, which causes them to release more silver ions and actively kill more bacteria. Because of their reduced size, easy penetration of these nanoparticles through the peptidoglycan layer of the bacterial cell wall damages the respiratory chain by interfering with the chain reaction. The zone of inhibition was calculated for dental pathogens against silver nanoparticles made from red algae. A larger zone of inhibition was observed at low concentrations. Additionally, low doses have greater therapeutic potential (Khan et al., 2022b).

*Syzygium aromaticum* (clove) has multiple applications as an analgesic and anesthetic in dentistry. It is also useful in the biogenic synthesis of silver nanoparticles. The resulting nanoparticles were found to be effective against various dental pathogenic microorganisms. Long-lasting bactericidal and bacteriostatic activity, biocompatibility, and minimal *in vivo* toxicity are the advantages of these biogenic nanoparticles (Jardón-Romero et al., 2022). An ethnobotanical substance, gum arabica, is derived from Acacia Senegal. The use of this herbal remedy to regulate the development of silver nanoparticles as components plays the role of capping and stabilizing agent in synthesis (Islam et al., 2021). The use of Arabic-moderated AgNPs is beyond the control of dental pathogens. These nanoparticles control SARS-CoV-2 viral infection. Both the oral cavity and the upper respiratory tract have been documented to be impacted by COVID-19. To eliminate respiratory-related viruses and the dental carcinogen S. mutans, AgNPs can be administered as a nasal spray or mouthwash (Al-Ansari et al., 2021). Ag-NPs of *A. vera* demonstrated antimicrobial potential against *E. faecalis*, *S. mutans,* and *C. albicans,* which are commonly associated with dental caries (Rodrigues et al., 2020).

### 6.6 Biosensing applications

The aggregation of gold nanoparticles enables the creation of colorimetric diagnostic techniques due to this abrupt color change. Semiconductor nanoparticles known as quantum dots (QDs) produced by zinc sulfide have applications in biosensing. *Aspergillus sp*. was used to create zinc sulfide (ZnS) QDs. It is a fungus with heavy metal tolerance and detoxifying routes found in fungi and the simplicity with which the entire biosynthetic reaction can be managed. Fungus systems would make great biofactories for the production of nanoparticles. The absorbance of ZnS QDs was examined in the absence and presence of heavy metals (Cd, Zn, Cr, and Pb). The absorbance maximum was found to be quenched in the presence of the heavy metal, resulting in a significant drop. Another aspect of the study also demonstrated the comparative efficiency of biogenic ZnS QDs over synthetic ZnS QDs (Jacob et al., 2019).

### 6.7 Anticancer applications

*Artemisia turcomanica* leaf extract is used in the green fabrication of silver nanoparticles (22 nm), and the gastric cancer cell line anticancer activity and induction of apoptosis are studied. The MTT test and biomass analysis findings demonstrated that the quantities of both commercial and photosynthesized AgNPs increased the inhibitory effect. Impact on the growth of AGS and L-929 cell lines. In comparison to commercial nanoparticles, photosynthesized AgNPs needed a lower dosage to limit cell development. According to research, silver nanoparticles interfere with signaling pathways and interact with membrane proteins to restrict cell development. Through fusion or endocytosis, nanoparticles enter a cell and disrupt the mitochondria’s electron transport chain (Mousavi et al., 2018).

The synthesis of silver palladium bimetallic nanoparticles (AgPd NPs) from aqueous *T. chebula* fruit extract is reported in this research study. These nanoparticles have significant anticancer potential against lung cancer cells (A549). *T. chebula* fruit extract contains glycosides, alkaloids, phenols, flavonoids, saponins, carbohydrates, steroids, reducing sugars, terpenoids, and proteins, which play an important role as capping and reducing agents in the synthesis of bimetallic AgPd NPs (Sivamaruthi et al., 2019). AgPd bimetallic nanoparticles have anticancer potential because they stimulate the formation of reactive oxygen species through respiratory chain dysfunction, which causes DNA damage. This triggers the apoptotic cascade pathway, which ultimately results in cell death (Sivamaruthi et al., 2019). NPs produced by microorganisms have excellent properties that make them effective against many cancer cells. Currently, to stop cancer cells from dividing, many pathways are used, such as chemotherapy, radiotherapy, immunotherapy, and therapy, but they all have some side effects, such as chemotherapy, which can develop resistance to drugs with 90% failure during metastasis. The synthesized particle size and monodispersity of the final product are influenced by the concentration of the raw materials, the bacterial strain, the metallic salts, and various components of the bacterial media. Nanomaterials can be used for cancer; they have multiple mechanisms of action to increase the effect and control and have become an alternative solution as anticancer agents. Microbial-synthesized NPs are being used to manage cancer in a green, ecologically benign, safe, and efficient manner. The primary raw materials for nanoparticles, metal ions, are trapped within or on the surface of microbial cells by electrostatic forces with enzymes such as reductases and NADH-dependent reductase that are both engaged in cellular processes and the formation of nanomaterials. Metal ions are among the harmful substances that may be removed from cells via efflux pumps. These metal ions can be handled by bacteria using energy-dependent ion effluxes. Biomineralization, biosorption, complexation, and precipitation are some of the pathways for the microbial synthesis of NPs. Size affects the anticancer effect. Smaller NPs are more effective for deep tumor tissue penetration. The caspase-3 enzyme was activated by the AgNPs, resulting in cytotoxicity to DLA cells and the induction of death. In the presence of AgNPs, the viability of the murine macrophage cell line and the human breast cancer cell line (MCF-7) was noticeably decreased. Human colon cancer and Dalton’s lymphoma (DL) cells had lower survival rates after exposure to the NPs. In research, AgNPs with a mean size of approximately 13 nm were produced using the actinobacterial strain SF23. magnetosomes, which are microbially derived NPs produced by magnetotactic bacteria. Magnetosomes are microbially derived NPs produced by magnetotactic bacteria. Iron-containing, lipid-bound nanocrystal structures are comparable to a polymeric shell, iron oxide core, and nanoparticles. Hepatic cancer cells were more effectively destroyed by doxorubicin-loaded magnetosomes. With applications in drug administration, immunostimulation, radiosensitization, and photothermal enhancement, NPs can also assist in other therapeutic modalities. Laboratory research has demonstrated the advantages of these nanoparticles in the administration of anticancer medications. Surprisingly, alteration of the drug surface affinity affects the rate of drug release by surface functionalization of these nanocarriers by increasing or lowering their surface hydrophobicity (Saravanan et al., 2021). Cervical cancer is one of the leading global causes of death in women. In this study, comprehensive information on the efficacy of biologically synthesized AuNPs against cervical cancer arising from cervix cells was given. According to the study, the ANPs treated cervical cancer by increasing intracellular ROS production and inducing apoptosis. The size of the AuNPs was found to be less than 100 nm. The metabolites in biological systems are crucial for the transformation of Au ions into AuNPs. Human cervical cancer cells have been found to overexpress folate receptors. According to research, adding folic acid to chemotherapy nanoformulations improved their ability to target cervical cancer cells. Applications of Biogenic Nanoparticles in biomedical science are summarized in Table 3.

**TABLE 3:** Applications of Biogenic Nanoparticles in biomedical science.

Nanoparticles	Material used	Applications	References
Anti-microbial applications			
Au	Banana peel extract	Activity against *E. coli*	SI et al. (2020)
Fe	Spinach leaf extract	Activity against *B. subtilis*	Tyagi et al. (2021)
Fe	Banana peel extract	Effective against *B. subtilis* and *E. coli*	Tyagi et al. (2021)
Cu	Magnolia leaf extract	Effective against *E. coli*	Rubilar et al. (2013)
ZnS	Aspergillus sp.	Effective against Gram-negative bacteria	Jacob et al. (2019)
Ag	Fenugreek seed extract	Effective against Gram-positive and Gram-negative bacteria	Awad et al. (2021)
Ag	Fruit pod of Cola nitida	Activity against *Klebsiella* granulomatis and *P. aeruginosa*	Lateef et al. (2016b)
Ag	Cinnamon tamala leaf extract	Effective against drug-resistant *E. coli* and *Klebsiella pneumoniae*	Shanmuganathan et al. (2018)
Ag	P. nigrum leaf extract	Antimicrobial activity	Augustine et al. (2014)
Ag	Fruit pod of Cola nitida	Activity against *A. fumigatus* and *A. flavus*	Lateef et al. (2016b)
Anti-Parasitic applications			
Ag	Agaricus bisporus isolate	Larvicidal action	Dhanasekaran and Thangaraj (2013)
Ag	*E. coli* isolate	Larvicidal action	Dhanasekaran and Thangaraj (2013)
Ag	Penicillium sp. Isolate	Larvicidal action	Dhanasekaran and Thangaraj (2013)
Ag	*Vibrio* sp. isolate	Larvicidal action	Dhanasekaran and Thangaraj (2013)
Ag	Extract of *L. aspera*	Mosquito larvicidal action	Elumalai et al. (2017)
Ag	Extract of H. suaveolens	Mosquito larvicidal action	Elumalai et al. (2017)
Ag	Extract of Cuminum cyminum	Anti-leishmanial activity	Bagirova et al. (2020)
Anti-Malarial Applications			
Ag	Gmelina asiatica leaf extract	Activity against *Aedes aegypti* and *Anopheles stephensi*	Govindarajan et al. (2016)
Ag	Drypetes roxburghii fruit extract	Mosquito biocontrol	Haldar et al. (2013)
Ag	Leaf extract of Camelia sinesis	Against Dental caries	Rajan et al. (2015)
Ag	Extract of Halymenia porphyriformis	Inhibit cavities and tooth decay	Khan et al. (2022b)
Ag	Extract of Solieria robusta	Inhibit tooth decay	Khan et al. (2022b)
Ag	Aqueous extract of Syzygium aromaticum	Strong antibacterial activity against oral pathogens	Jardón-Romero et al. (2022)
Ag	Extract of Acasia senegal	Against Dental carcinogen and respiratory-related viruses	Al-Ansari et al. (2021)
Anticancer applications			
Ag	Leaf extract of Artemisia turcomanica	Anticancer activity (Gastric)	Mousavi et al. (2018)
AgPd	Aqueous Terminalia chebula fruit extract	Anticancer activity (Lung)	Sivamaruthi et al. (2019)
Other Applications			
Cu	Extract of Calotropis procera L	Antioxidant activity	Rubilar et al. (2013)
ZnS	Aspergillus sp.	Detection of Hg, Cu, and Mn ions	Jacob et al. (2019)

**FIGURE 7:**
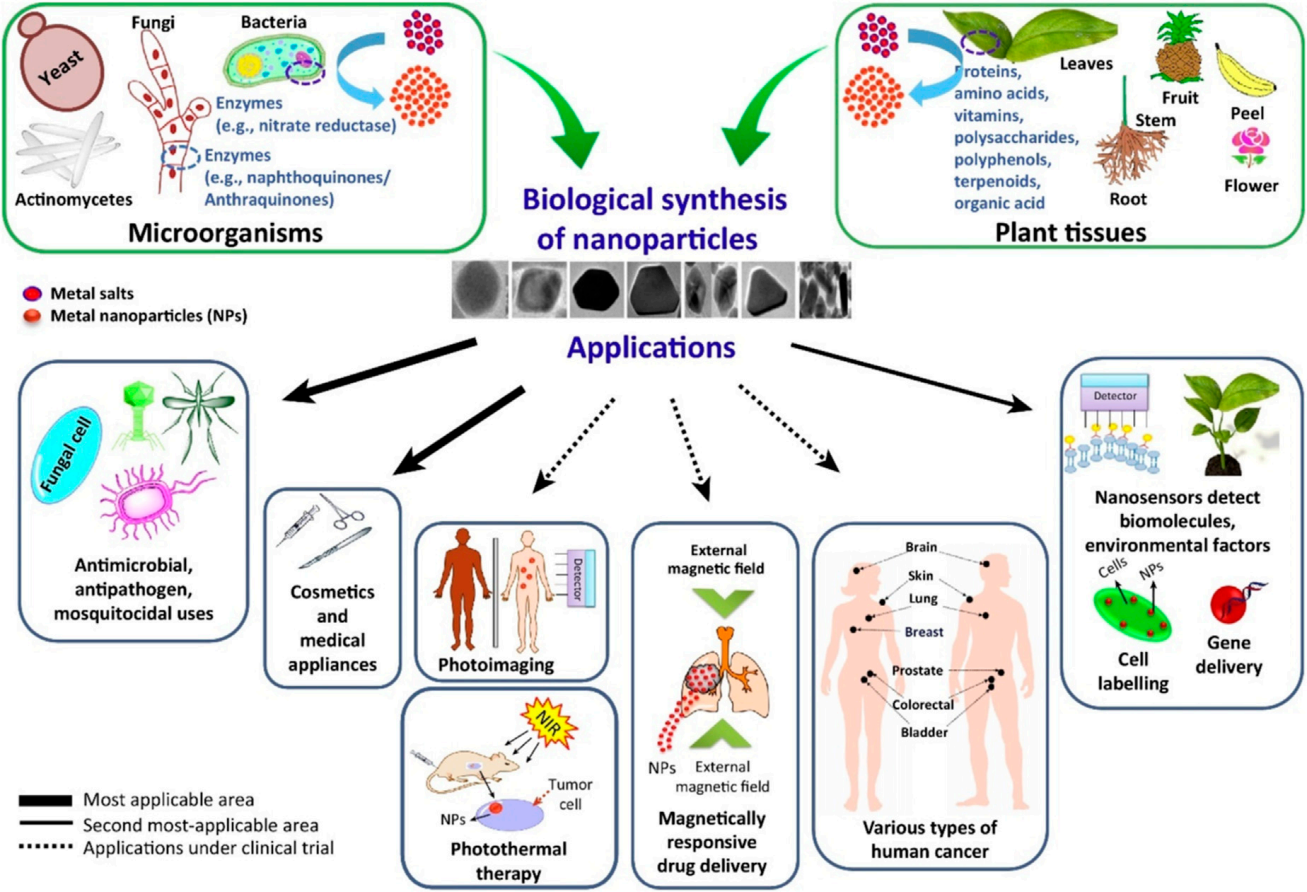
Summary of various applications of metal nanoparticles in biomedical and environmental fields. Adapted from Ref. (Singh et al, 2016).

In recent times, researchers have focused on the synthesis of gold nanoparticles by biological as well as chemical methods toward cancerous (HeLa, MCF-7, A549, and H1299) and normal (HEK293) cell lines with the comparative analysis of biologically synthesized gold nanoparticles using extracts of the tulsi (*Ocimum sanctum*) plant with chemically synthesized citrate-capped particles for their anticancer behavior. Chemical reduction is the most frequently used method for the synthesis of gold nanoparticles. Trisodium citrate was used as a reducing agent to synthesize citrate-capped GNPs. The Brust–Schiffrin method was employed for two-phase synthesis by chemical modes, and leaf extracts of the tulsi (*Ocimum sanctum*) plant have been used for biogenic synthesis, which has excellent bioreduction properties due to its metabolite constituents. A comparative evaluation of the anticancerous properties of biogenic and chemically synthesized GNPs was performed by ROS determination, DNA fragmentation, mitochondrial functions, protein extraction, and western apoptosis. Biogenic GNPs were found to be more cytotoxic on cancerous cells than chemical GNPs in cell proliferation and IC50 determination; additionally, in the case of normal cells, bGNPs were comparatively less cytotoxic and potentially nontoxic toward HEK293 cells based on the MTT assay (Virmani et al., 2020). Significant applications of biogenic nanomaterials in cancer theranostics are illustrated in Figure 7 (Singh et al, 2016).

### 6.8 Drug delivery applications

Nanoparticles synthesized from biological sources are prime nanocarriers for drug delivery. The modulation of their physical properties makes them suitable candidates for drug delivery (More and Deshmukh, 2020). Large biomolecules may be delivered by gold nanoparticles. They serve as an effective scaffold for the identification and distribution of biomolecules due to their functional versatility and tunable size (Sharifzadeh and Hosseinkhani, 2017). Peptides, proteins, and nucleic acids such as DNA and RNA have all been delivered successfully. By using noncovalent conjugation, gold nanoparticles for nucleic acids may be delivered. They may produce a high surface-to-volume ratio, enhancing the payload/carrier ratio. Gold nanoparticles functionalized with cationic quaternary ammonium groups are bound to plasmid DNA by electrostatic interactions, and the bound DNA is then released by GSH treatment. In mammalian 293T cells, these noncovalent DNA-nanoparticle conjugates offer an efficient method of gene delivery. Using covalent bonding, gold nanoparticles can carry nucleic acids. To graft onto nanoparticles, thiols (-SH) might readily modify nucleic acid strands. To transport siRNA that has been thiolated (SH-siRNA) to cells, Nagasaki and others coupled it with gold nanoparticles. Endocytosis is triggered by the adsorption of many serum proteins onto the surface of the DNA-NP particle (Nagasaki, 2008). The ability of functionalized gold nanoparticles to transport insulin was recently established by Pokharkar, Sastry, and others (Bhumkar et al., 2007). Chitosan is a biopolymer that stabilizes particles. The distribution of insulin across mucous membranes is made possible by chitosan-coated particles, which firmly bind insulin to their surfaces (Ghosh et al., 2008). Biogenic nanoparticles have widespread applications in the area of biomedical sciences. Metallic nanoparticles synthesized from biogenic sources are used for theranostic as well as drug delivery purposes. It is one of the trending areas of research in green nanotechnology. Biogenic nanocarriers are a trending area of research worldwide. Various patents are filed for research based on the biogenic synthesis of nanomaterials for biomedical applications. Table 4 illustrates the various patents and patent applications in the proposed area.

**TABLE 4:** The patent scenario of biogenic nanoplatforms.

Patent number	Description	Biomedical applications
US9403688B1	Method for preparing biogenic silica nanoparticles from seed hulls of cultivated plants	Biosilica is a selective inducer of osteoprotegerin, which results in the inhibition of osteoclast differentiation and activation that results in reduced resorption of bone. It’s also used for various other purposes such as thermal insulation, pesticides, food additives, drug delivery, gene therapy, dietary supplement for women to increase bone mineral density via osteoblast differentiation
EP2671450A1	Method of preparing nanoparticles from the extract of algae	Silver Nanoparticles obtained by this method demonstrate antibacterial potential against both Gram-negative and Gram-positive bacteria and can be used in household items, food packaging, cosmetics, and pharmaceuticals
WO2013143017A1	Synthesis of gold nanoparticles from fungi Botrytis Cineria	Photoluminescence, medical diagnosis, used in therapy against some types of cancers
US20100055199A1	Synthesis of silver nanoparticles from Trichoderma fungi	Used as an antibacterial against various types of bacteria, fungi, and viruses
WO2016106466A1	Synthesis of gold nanoparticles from plant extracts obtained from seeds, leaves, stem, fruit, and flower	Medical diagnosis imaging, solar energy conversion, semiconductor manufacturing, catalysts, water treatment, and in the therapy of some types of cancers
US20160263657A1	Preparation of stable metal nanoparticles in a one-step process	This procedure has the uniqueness of producing extremely small metal nanoparticles which allows them to be dispersed in water for after 6 months at room temperature. Since these nanoparticles are made in an aqueous solution, it provides a greater range of applications in the field of diagnostics, medicine, *etc.*
WO2011041458A1	Preparing metal nanoparticles from solutions of fruit extract (juice, pulp, skin, seed *etc.*)	Removing contaminants from soil and groundwater—they can act by chemical or biological reduction process or a combination of both to detoxify the contamination. They can be used for catalysis, wastewater treatment, and water treatment
CN103949658A	Synthesis of silver nanoparticles from the aqueous extract of bark of eucommia ulmoides	Bioanalytical chemistry, industrial catalysis, food security checks
US9789146B1	Synthesis of nanoparticles from the plant powder of adansonia digitata	Recommended for pregnant women due to its high content of vitamin C. Additionally, used as a hepatoprotective, antiviral, antimicrobial, antioxidant, hypoglycemic and anti-inflammatory agent
US10856559B1	Procedure for producing eggshell derived nanoparticles	Useful as an anticancer agent for human breast cancer and lung cancer
US9700512B1	Preparation of Hesperetin nanoparticles	Treatment of lead-induced oxidative stress in various organisms
US9974750B1	Method for the preparation of ifflaionic acid nanoparticles	It possesses potent antitumor activity against lung cancer cells, cervical cancer cells, human colon cancer cells
US10947266B2	A method for the synthesis of ursolic acid nanoparticles	Anticancer, anti-inflammatory, immunomodulatory activity. Antibacterial against Gram-positive and Gram-negative bacteria and fungi
CN103406548A	Obtaining silver nanoparticles from tapioca starch	Controls pathogenic bacteria (*S. aureus* and *E.* Coli) and acts as a disinfectant
US6783963B2	Preparation of metal sulfide nanoparticles using fungi (Fusarium Oxysporum sp.)	Used as markers in drugs, catalysis, gene sequencing
CN106513707A	Preparation of silver nanoparticles from the extract solution of leaves of blueberry	Bacteriostatic, fungistatic, reducing agent
KR102292488B1	Manufacturing of silver nanoparticles from Citrus fruits	Used in cosmetics, quasidrugs, food, catalysis, and sterilization processes. Additionally, used as an antibacterial agent
US20050009170A1	Preparation of metal nanoparticles in plants	Used for Catalysis. Used for making water-resistant products (sunscreens)

## 7 Disadvantages of biogenic metallic nanoparticles

A wide variety of metallic and metallic oxide nanoparticles are synthesized by applying green nanotechnology. Different applications of these nanoparticles are investigated after studying the limitations and issues that these synthesized nanoparticles pose to human health. The main issues with environmentally friendly synthesized nanoparticles are related to biosafety, cytotoxicity, difficulty in controlling the shape and size of the produced nanocomposites, and purification. Nanoparticles produced by different mechanisms produce nanoparticles of different sizes. In many organisms, such as fungi, the intracellularly yielded particles are smaller than those fabricated extracellularly. In this case, it is difficult to create monodisperse nanoparticles (Pantidos, 2014). Biosafety is also a crucial concern about biogenic nanoparticles. The use of viruses, certain fungi, and bacteria can be dangerous to human health, as it can lead to an immunogenic response. Viral nanocomposites from the hepatitis B virus (Steinmetz et al., 2009) and bacteriophages (Prasuhn et al., 2008) and from fungi *F. oxysporum* (Syed and Ahmad, 2012) are the most likely candidates for such immunogenic reactions. Similarly, cytotoxicity is one of the biggest drawbacks of metallic nanoparticles and a prime consideration for nanotechnology. Nanotoxicology is a branch to study the long-term exposure effect of these particles on human tissue. Studies suggest that half of the cytotoxicity concerns are the result of vulnerability to metallic nanoparticles (Wang et al., 2012). Metallic oxide nanoparticles tend to aggregate inside cells and around organelles, interfering with their normal function and disturbing homeostasis (Iavicoli et al., 2012). Nanotoxicological reports of a wide range of metallic oxide nanoparticles have shown that nanocomposites such as copper oxide (CuO), silica nanoparticles, cobalt oxide (Co_3_O_4_), iron oxide (Fe_2_O_3_), zinc oxide, and titanium dioxide nanoparticles (TiO_2_) are capable of inducing toxic reactions in human cells. When CuO nanomaterials were exposed to the human lung epithelium and evaluated by Comet assay, oxidative lesions and damage to epithelial DNA were observed (Karlsson et al., 2008). Additionally, bacterial magnetite was found to induce ROS-induced oxidative stress (Wu et al., 2014). A study conducted on silica nanoparticle-induced nanotoxicity showed the effect of the release of reactive oxidative species adding to apoptosis (McCarthy et al., 2012). Similarly, a cytotoxic effect was also shown by zinc oxide nanoparticles synthesized from the leaf extract of *Tabernaemontana divaricate*, and this effect was seen in MCF-7 breast cancer cells (Sivaraj et al., 2014).

Other than the composition of the nanomaterials, many other things govern the toxicity to humans and other living systems, such as the shape, size, and dose of nanoparticles administered. A high dose is associated with increased cytotoxic effects. To make these biogenically produced nanoparticles safe and effective, it is important to perform nanotoxicological investigations and study the mechanisms of cytotoxicity to standardize the toxicity protocols.

## 8 Factors influencing the biological properties of biogenic metallic nanoparticles

The biological properties of biogenic nanoparticles are affected by several critical factors, such as size, shape, surface coating, and surface charge. The permeability characteristics as well as interaction at the biological interface differ based on variabilities in their morphology. The crystalline structure of biogenic metallic nanoparticles is slightly different from that of chemically synthesized metal nanoparticles. The change in crystalline nature progressively increases the percentage of permeability for biogenic metallic nanoparticles. The morphological characteristics include size, shape, surface texture, *etc.* For biomedical applications, a size of less than 100 nm is desirable to provide a prompt biological response (Patil et al., 2018). Metal nanoparticles have a size below 50 nm, which is effective for applications. Another disadvantage associated with metal ions is that deposition may deteriorate normal cell behavior. Chemically synthesized metal nanoparticles have higher deposition in normal cells than biogenic metal nanoparticles. Even the rate of elimination of biogenic nanoparticles is high due to their unique size and surface characteristics. The PEGylated NPs provide high blood circulation time Virmani et al. (2020). Compared the performance of chemically synthesized metal nanoparticles and biogenic nanoparticles on cancer cell lines (HeLA, MCF-7, A549, H1299, and HEK293). The size of biogenic nanoparticles (2–10 nm) was slightly less than that of chemically synthesized nanoparticles (5—20 nm). The lowest particle size shows high inhibition potential against cancer cell lines. Chemically synthesized nanoparticles have less cytotoxic effects than biogenic nanoparticles with relatively lower relative oxygen generation capacity. Many scientists utilize surface engineering approaches to modulate or add features to the physicochemical and biological behavior of nanoparticles. The surface charge of bare or modified nanoparticles principally affects the stability of metal ions in dispersion media. The highly negatively charged or positively charged ions repel each other and form a stable dispersion. However, in the case of the metal core, they start aggregating in the solution and are suspended at the bottom of the dispersion. Additionally, the surface charge of metal ions shows variable absorption at the biological interface. Different grades of surfactants, biopolymers, and natural extracts are used to coat or encapsulate biogenic nanoparticles. In most cases, the *in situ* synthesis protocol leaves the coating over biogenic nanoparticles and acts as a stabilizer. *Spinacia oleracea* extracts show synergistic therapeutic potential during the synthesis of biogenic silver nanoparticles. The extract possesses good anticancer and antioxidant activity and acts as a capping agent for silver nanoparticles. Biogenic silver nanoparticles show no toxic effect, but the presence of *S. oleracea* leaf extracts shows high performance against leukemia cells (Zangeneh, 2020). Plant extracts act as reducing and capping agents and promote therapeutic outcomes in cancer therapy. Multiple biomolecules serve as stabilizing and capping agents during the synthesis of NPs, and greenly generated metallic NPs exhibit stronger antibacterial potential than conventionally manufactured NPs (Bahrulolum et al., 2021). The effect of capping agents on biogenic nanoparticles and their therapeutic potential are extensively elaborated in a recent review[219].

## 9 Conclusion and future perspectives

Nanoparticles are produced through several physical and chemical processes, but these techniques have drawbacks such as high prices and the need for high temperature and pressure. Much focus is now shifted to recent advances in nanotechnology and the environmentally sustainable manufacturing of nanomaterials using plants and microorganisms. Biosynthesis procedures for nanoparticles are rapid, simple, inexpensive, eco-friendly, safe and impart high stability with efficiency. Green nanoparticles can help sustainable development because they can be synthesized using renewable materials and can be easily recycled, reducing the environmental impact of nanoparticle synthesis and waste formation. Scientists have focused their energy on natural resources because of their abundant nature, environmental friendliness, scale-up, and affordability. The green synthesis of nanoparticles has many advantages, such as the use of nontoxic solvents, efficient production processes and microbiome-friendly synthesis. The synthesis parameters influence the production yield of most metallic nanoparticles. However, most studies use extracts or solvent compositions without proper quantitation of each component to create a nonreproducible synthetic route that is difficult to scale up. Furthermore, the species type creates variable extracts that are impossible to control in unit operations. The biostability of the nanoparticles depends on their surface charge and shape, and the charge is influenced by enzymes and vitamins in the production medium. We observed that the studies typically follow synthesis, characterization, and *in vitro* evaluation on either cell lines or microbes. Some researchers have demonstrated wound-healing abilities but have often used topical applications. The percentage of reports with *in vivo* particle kinetics and biodistribution profiles is dismal, and this is an essential criterion for regulatory approval for most nanomedicines. Additionally, degradation pathways need special focus, just demonstrating *in vitro* degradation is not sufficient. In certain conditions, simulation studies are useful to a limited extent. Although the green synthetic production and biomedical applications of biofabricated nanoparticles are exciting, the discussed issues need special attention in future studies.
